# Supplementary material for: Risk of myocarditis and pericarditis after a COVID-19 mRNA vaccine booster and after COVID-19 in those with and without prior SARS-CoV-2 infection: A self-controlled case series analysis in England
Source: PLoS Med. 2023 Jun 7;20(6):e1004245. doi: 10.1371/journal.pmed.1004245 (PMC10286992; doi:10.1371/journal.pmed.1004245)
Supplement: S1 Protocol — (PDF) [file pmed.1004245.s002.pdf]

Protocol: Analysis of hospital admissions and emergency care consultations for acute myocarditis and pericarditis after COVID-19 vaccines in England

Immunisation Department, UK Health Security Agency

Version number 5.4

19/07/2022

1) Background

Reports of myocarditis clustering in the week after receipt of the Pfizer BioNTech BNT162b2 mRNA vaccine in Israel suggested a potential causal association, particularly in young males after a second dose. [1]

A matched cohort analysis using data from a large Health Maintenance Organisation (HMO) in Israel showed an elevated incidence of myocarditis after the BNT162b vaccine [2] although this was lower than after SARS-CoV-2 infection. A subsequent analysis of the same Israeli HMO data base showed that the vaccine-associated risk was highest in males aged 16-29 years within 6 days of the second dose. [3]

In Israel only the BNT162b2 vaccine is in use so the risk after other vaccines such as the AstraZeneca ChAdOx1 adenovirus vector vaccine or the Moderna mRNA-1273 could not be assessed. A case series study in the US, where both mRNA vaccines are used, suggested that the risk was associated with the mRNA vaccine platform, rather than a specific manufacturer's product. [4] mRNA vaccine-associated myocarditis cases are reported to be mild and usually recover within 1 to 3 weeks of onset. [5]

In the UK, three vaccines, ChadOx1, BNT162b2 and mRNA-1273, are in use in the national Covid-19 immunisation programme. Yellow Card reports to the UK Medicines and Healthcare products Regulatory Agency (MHRA) show a higher reporting rate after the two mRNA vaccines than the adenovirus vector vaccine for myocarditis and pericarditis, particularly among males after a second dose.[6] On 23rd August 2021 the MHRA issued information for health care professionals and vaccinees on the risk of myocarditis and pericarditis following Covid-19 vaccination, specifically mentioning the signal after BNT162b2 and mRNA-1273 vaccines. [7] MHRA advised that, since myocarditis and pericarditis can present with new onset of chest pain, shortness of breath or feelings of having a fast-beating, fluttering, or pounding heart, anyone who develops these symptoms within 2 weeks of a COVID-19 vaccination should urgently seek medical assistance.

Advice from the Joint Committee on Vaccination and Immunisation (JCVI) issued on 15th November 2021 was that those aged 16-17 years should receive a second dose of an mRNA vaccine as the overall risk benefit was favourable not withstanding the rare risk of vaccine- associated myo/pericarditis. [8] JCVI also said that an extended dose interval (as used in the UK) may reduce the risk of myocarditis from the second vaccine dose, although a causal association between dose interval and risk from myocarditis following vaccination has not yet been established.

A preliminary analysis of cases of myocarditis and pericarditis admitted to National Health Service (NHS) hospitals in England using the Secondary Users Service (SUS) indicated a low rate of admission after Covid-19 vaccines and no clear signal of an increased risk associated with the BNT162b2 vaccine. Since it is likely that many individuals presenting with the symptoms described by the MHRA will not be admitted, analysis of cases presenting to emergency care in England who are diagnosed with myo/pericarditis may be a more sensitive method for identifying cases. The troponin test that is used to diagnose myocarditis can usually be completed rapidly, so diagnosis of myocarditis can be made in the emergency care setting. Similarly for pericarditis for which diagnosis can be made in the emergency room on the basis of symptoms, auscultation and ECG findings.

The current study will assess whether there is an increased risk of presentation to emergency care, or hospital admission, with myocarditis or pericarditis after any of the three Covid-19 vaccines used in England. The risk of acute myocarditis or pericarditis after a laboratory confirmed SARS-CoV-2 infection will also be assessed.

2) Data sources

ECDS

The Emergency Care Data Set (ECDS), which is the National Health Service (NHS) data set for urgent and emergency care in England , will be used for case ascertainment [9], supplemented by hospital admitted cases in SUS that are not identified in the ECDS data set. Diagnoses in ECDS are assigned using the SNOMED coding system [10]. Preliminary analysis indicate that of the 14 codes that could indicate myocarditis or pericarditis, 98.6% were assigned to one of two codes. These were SNOMED code 50920009 for myocarditis (15.84%) and 3238004 for pericarditis (82.73%). The analyses will therefore be restricted to ECDS diagnoses with one of these two codes in the first two diagnosis fields. The ECDS data are timely and are near complete within a week from event date.

SUS

A second set of outcome events based on in-patient admissions in the Secondary Users Service (SUS) database for England for the same time period will also be used for case ascertainment. The SUS database provides ICD-10 coded diagnoses for cases admitted to all National Health Service hospitals in England within a few weeks of an admission. The following codes in any of the first three diagnostic fields will be used to identify admissions for myocarditis and pericarditis: I30 acute pericarditis, I40 acute myocarditis and I51.4 myocarditis, unspecified.

NIMS

Immunisation data will be obtained from the National Immunisation Management System (NIMS) using NHS number. The NIMS database of Covid-19 vaccine-eligible individuals in England was constructed using NHS numbers of individuals identified in electronic NHS records [11]. Since the NHS is the universal health care provider in the UK free at the point of access to all UK residents it has near complete coverage of the resident population. The NIMS denominator population is updated weekly to reflect known changes in the resident population including deaths. Daily updates of vaccinations (date, batch number and manufacturer) given to the eligible population are provided by NIMS.

SGSS

The results of SARS-CoV-2 tests carried out in England are collated in SGSS. This data set will be used to identify SARS-CoV-2 infections in the study population.

3) Analytic approaches

Two analytic approaches to assess the risk associated with vaccination will be used. First a retrospective cohort analysis will be conducted in which the incidence of the outcomes of interest in pre-specified post-vaccination periods will be compared with that in unvaccinated individuals after adjustment for available confounding factors. Second, a self-controlled case series (SCCS) analysis will be undertaken in vaccinated individuals with the outcome event to assess the incidence in pre-specified post-vaccination risk periods compared with the incidence outside these risk periods. [12] The SCCS analysis implicitly controls for any non-time varying factors not included in the cohort analysis that are associated with both vaccination and the outcome event.

Based on the onsets of the cases described in the epidemiological studies and case series reports, the risk periods are defined as 0-6 days and 7-13 days after date of vaccination, with the event date corresponding to the date of ECDS attendance or SUS admission.

In the SCCS analysis the risk of acute myocarditis or pericarditis after a SARS-CoV-2 infection will also be assessed. Multiple SARS-CoV-2 infections per person will be included in the analysis, and a new infection will be defined as a positive test at least 90 days after a previous new positive test.

4) Cohort analysis

a. Study population

The study population for the retrospective cohort will comprise the resident population in England who are registered on the NIMS database as eligible to receive a COVID-19 vaccine and are aged 12 years or above as at 31st August 2021 (the date now used by NIMS for assigning age). In addition to demographic information, (age, gender, ethnic group) the NIMS denominator uses information from primary care and other electronic health records to flag those in a clinically extremely vulnerable (CEV) risk group (available in NIMS since 8th December 2020) and those with a wider range of co-morbidities (available in NIMS from week 7 2021). The conditions included in the CEV category (now termed high risk) and those included in the wider group of those at risk are listed in the Department of Health Green Book chapter 14a on Covid vaccination. [13] Also flagged in NIMS are health and social care workers (under 65 year olds only) but this flag will not be used as preliminary analysis has shown that being a health or social care worker is not a confounder.

b. Outcome events

ECDS consultations in individuals between week 8 2020 (starting 22nd February 2021) and the end of week 5 2022 (6th February 2022) with a SNOMED code of 50920009 or 3238004 in the first two diagnosis fields will be identified. The start date for the study period of 22nd February 2021 will allow inclusion of the other risk group flag for all individuals in the study cohort. The following data items in the ECDS record will be extracted: SNOMED codes, gender, ethnicity, age at event date and discharge information (ie whether the consultation was closed, or the patient referred onwards or admitted for in-patient care). The ECDS data will be extracted at least a week after the study end date to accommodate the time lag before ECDS data are generally complete. The study end date may be extended if the workload of the study team precludes carrying out an ECDS data extract at the planned time.

Similarly, the second set of SUS outcome events (ICD-10 code in first 3 diagnosis fields: I30 acute pericarditis, I40 acute myocarditis and I51.4 myocarditis, unspecified) based on in-patient admissions for the same time period will be extracted. The SUS data will be extracted at least three weeks after the study end date to accommodate the time lag before SUS data are generally complete.

ECDS and SUS data will be analysed separately, representing those presenting with any indication of an event and those hospitalised, respectively. The first occurrence in the study period and without a prior ECDS consultation or SUS admission for myocarditis or pericarditis since 1st December 2019 will be identified.

c. Data linkage

The NIMS and ECDS/SUS data sets of outcome events will be linked on NHS number using the field NhsNumberValid in ECDS and FinalDigitschecksum in SUS to exclude those without a valid NHS number.

Those ECDS/SUS records that do not link will be excluded from the analysis. For the linked records the numbers with missing variables that are used in the adjusted analyses will be listed.

The ECDS/SUS data set of outcomes will also be linked to the Pillar 2 testing database on NHS number and date of birth. Testing data will be linked to NIMS demographic file to augment the National Health Service number in the testing data using combinations date of birth, surname, first name, and postcode using deterministic linkage with >95.5% uniqueness.

d. Construction of the study cohort

Cumulative counts of the NIMS data extract will be stratified by vaccination status, age at 31 Aug 2021 (12-15, 16-17, 18-19 then 5-year bands), gender, ethnic group, region, CEV, and other clinical risk group. Ethnic group in NIMS is based on 17 categories [14] and for other UKHSA analyses have been collapsed into five main groups (White, Mixed or

Multiple ethnic groups, Asian or Asian British, Black, African, Caribbean or Black British)[1] plus a not known group. For those that are vaccinated, the post-vaccination time will be stratified by 0-6 days, 7-13 days and 14+ days after a first, second dose or third dose, and separately for ChAdOx1, BNT162b2 and mRNA 1273 vaccines (and for these primary schedules combined with the BNT162b2 and mRNA 1273 boosters). At the time of the event those who have received a mixed primary schedule, first and second doses <19 days apart, or second and third doses < 56 days apart, or a third ChAdOx1 dose, or a 3rd dose before 1st September 2021 or a 1st dose before 8th December 2020 will be excluded from cases and the denominator, as will any recipients of other Covid-19 vaccines which may have been given as part of a vaccine trial.. Only those with complete data will be analysed.

Outcome events stratified by the same factors will be merged to obtain a data set of counts and population denominator by day, age group, gender, region, ethnic group, CEV and other clinical risk group. Repeat consultations for myocarditis or pericarditis within 42 days of an earlier event will be excluded.

e. Statistical analysis

Descriptive: Myocarditis and pericarditis cases will be described by week, age group, gender and ethnicity, with each stratified by vaccination status. The proportions in each category admitted for in-patient case will be given. In addition person time will be tabulated by outcome event, vaccination status (unvaccinated or vaccinated by dose and vaccine type), age group (12-29, 30-39,40-64,65+), gender, ethnic group, time period, CEV and other clinical risk group.

Statistical model: Poisson regression will be used to model the data with an offset for population at risk (person days). Adjustment for covariates will be by age group (12-15, 16-17, 18-19 then 5 year bands), gender, ethnic group, region, CEV, other clinical risk group, and 4-week interval. The core model will be with an age stratification of 12-15, 16-39, ,40+, fully adjusted as well as all ages combined, and separately with a gender stratification.

The following sensitivity analyses will be conducted.

i) Restricting outcome events to those before 23rd August when MHRA issued the information about symptoms associated with myocarditis though it is recognised that this will likely lack power to detect an elevated risk in the youngest age group as under 30 year olds who were not HSCWs and had no clinical risk only became eligible for vaccination in the second week of June 2021.

f. Significance and multiple testing

Relative incidence (RI) will be shown with 95% confidence intervals. Given the relatively large number of risk intervals, and vaccine type/number of doses that will be examined, significance will be assessed at a 1% and 0.1% level as indicated as * =P<0.01 and ** = P<0.001. Relative incidence estimates will not be calculated where the number of events in the period is 0 or 1. Also where the number of events is 2 or 3 this will not be regarded as strong evidence irrespective of significance.

g. Attributable risk

This will be calculated based on the attributable fraction AF= (RI-1)/RI multiplied by the number of cases. The RI will be calculated across any period of increased risk (i.e 0-6, 7-13 or 14+). The AF will be applied to the events to estimate attributable events (AE). To convert to attributable risk (AR) per dose the person time in years in the period of interest

---

[1] The five main groups comprise the following subgroups in NIMS
White includes : English, Welsh, Scottish, Northern Irish or British, Irish, Gypsy or Irish Traveller, Any other White background
Mixed or Multiple ethnic groups includes: White and Black Caribbean, White and Black African, White and Asian
Asian or Asian British includes: Indian, Pakistani, Bangladeshi, Chinese, Any other Asian background
Black, African, Caribbean or Black British includes: African, Caribbean, Any other Black, African or Caribbean background
Other ethnic group includes: Arab, Any other ethnic group.

will be converted to doses with follow-up for the interval of interest by dividing person time in days by length of period (so for example for a 7 day window Doses = p-yrs * 365 / 7). AR = AE / Doses.

5) Self-controlled case series analysis (SCCS)

A SCCS analysis will be conducted and will include both Covid-19 infection, as this may cause myocarditis or pericarditis [2], and vaccination as separate exposures in the same model.

For assessing vaccination as an exposure risk for myocarditis/pericarditis, all first ECDS/SUS outcome events in the study period (22nd February 2020 to 6th February 2022) which have linked to an individual aged 12+ who had received a primary dose of ChAdOx1, BNT162b2 or mRNA-1273 vaccine before or after the date of myocarditis/pericarditis outcome event will be included in the SCCS analysis. We will exclude all those who have received a mixed primary schedule, first and second doses <19 days apart, or second and third doses < 56 days apart, or a third ChAdOx1 dose, or a 3rd dose before 1st September 2021, or a 1st dose before 8th December 2020. Any recipients of other Covid-19 vaccines that may have been given as part of a vaccine trial will also be excluded. The risk periods for vaccine as an exposure will be 0-6 and 7-13 days post each dose of vaccine and vaccine type.

For assessing SARS-CoV-2 infection as an exposure risk for myocarditis/pericarditis, the exposure date will be the date of test in SGSS; the outcome event date will be the same as for the cohort analysis (ie ECDS consultation date or SUS admission date). The risk period to be used for infection is more difficult to define as some individuals may not present with myocarditis or pericarditis until some weeks after the start of the SARS-CoV-2 infection when they develop this as a late complication, while others may seek a test because of the onset of shortness of breath associated with the cardiac complication rather than the symptoms associated with the initial respiratory tract infection. A longer risk period of 0-27 days after symptom onset/PCR test date will therefore be used. Those whose positive test was on the day of ECDS attendance (interval 0 days) will comprise a separate risk interval. SGSS will be linked with the ECDS/SUS data extract to identify those with a myocarditis/pericarditis outcome event in the study period occurring before or after a confirmed SARS-CoV-2 infection.

Occurrence of one of the outcomes of interest after a first dose is not stated as contraindication to receipt of a second dose in section 4.3 of the mRNA SmPCs (BNT162B2 available at [15] and mRNA-1273 available at [16]). However, myocarditis and pericarditis after mRNA vaccination is listed under Section 4.4 Special Warnings and Precautions for Use, so those with an event after a first or second dose may be advised not to get another dose. If so this would violate the assumption behind the SCCS approach that vaccination is not dependent on the occurrence of the event. However, it is expected that most cases will go on to complete the full vaccination schedule. We propose that the main analysis will account for short-term dependence only, but longer-term dependence will need to be investigated. Firstly, the length of the pre-exposure interval will be varied and the relative incidence 0-13 days post exposure and pre-exposure relative incidence will be plotted against length of pre-exposure interval. If the relative incidence 0-13 days post exposure plateaus with increasing length of pre-exposure interval, this will indicate that event dependence is short-term. Secondly, two sensitivity analyses will be carried out taking only those individuals with no record of infection (1) using the standard SCCS method and (2) using the event-dependent adaption of the SCCS method developed by Farrington et al.[17] (as used in Stowe et al.[18]) to accommodate the contraindication of further vaccination after a prior post-vaccination event. Any appreciable discrepancy between the two sets of results from (1) and (2) will be taken as an indication that contraindication is leading to bias.

Assuming no or negligible bias is found relating to long-term event-dependence, the main analysis will proceed as a standard SCCS. Baseline incidence will be the period before and 14+ days after each dose, or before or 28+ days after a positive SARS-CoV-2 test. To accommodate the temporary deferral of vaccination while the individual is recovering from the event a pre-vaccination risk period of 21 days will be removed from the baseline. Similarly person time for 14 days prior to SARS-CoV-2 onset/Pillar 2 test date will be removed from the baseline in case the ECDS consultation resulted in exposure to Covid-19 over and above that in the community, or onset is unknown. Period adjustment will be included with period in 4-weekly intervals. SCCS analyses will include (i) all ages 12+, (ii) restricting to ages 16-39, (iii) ages 40+, (iv) ages 12-15 if there are sufficient case numbers, (v) males only, (vi) females only. Rules for significance and multiple testing outlined for the cohort analysis will also be applied to the SCCS.

The following sensitivity SCCS analyses will be carried out:

i) starting the observation period at first SARS-CoV-2 infection and not censoring at death
ii) restricting the observation period to end on 23rd August 2021

SCCS post-hoc analyses (19/07/2022)

After all analyses had been carried out, we noticed that our myocarditis RI for 1-27 days post SARS-CoV-2 was lower in our analysis than that published in Panone et al. [19], which was similar to our analysis except that an earlier period was covered and only the first positive SARS-CoV-2 test was included. We will therefore take all individuals with either a positive SARS-CoV-2 test or a record of vaccination (i.e. unvaccinated but infected will be included in addition to the main analysis), and will carry out 3 additional analyses that separate the 1-27d risk window following a positive SARS-CoV-2 tests within our analyses by: (1) by each variant-dominant period, (2) first and subsequent positive tests and (3) pre- and post- vaccination positive tests.

References

1) Mevorach D, Anis E, Cedar N, Bromberg M, Haas EJ, Nadir E, et al. Myocarditis after BNT162b2 mRNA Vaccine against Covid-19 in Israel. N Engl J Med. 2021 Oct 6:NEJMoa2109730. doi: 10.1056/NEJMoa2109730. Epub ahead of print.

2) Barda N, Dagan N, Ben-Shlomo Y, Kepten E, Waxman J, Ohana R, et al Safety of the BNT162b2 mRNA Covid-19 Vaccine in a Nationwide Setting. N Engl J Med. 2021 Sep 16;385(12):1078-1090. doi: 10.1056/NEJMoa2110475. Epub 2021 Aug 25.

3) Witberg G, Barda N, Hoss S, Richter I, Wiessman M, Aviv Y et al Myocarditis after Covid-19 Vaccination in a Large Health Care Organization. N Engl J Med. 2021 Oct 6:NEJMoa2110737. doi: 10.1056/NEJMoa2110737. Epub ahead of print.

4) Perez Y, Levy ER, Joshi AY, Virk A, Rodriguez-Porcel M, Johnson M et al.. Myocarditis Following COVID-19 mRNA Vaccine: A Case Series and Incidence Rate Determination. Clin Infect Dis. 2021 Nov 3:ciab926. doi: 10.1093/cid/ciab926. Epub ahead of print.

5) Das BB, Moskowitz WB, Taylor MB, Palmer A. Myocarditis and Pericarditis Following mRNA COVID-19 Vaccination: What Do We Know So Far? Children (Basel). 2021 Jul 18;8(7):607. doi: 10.3390/children8070607.

6) Coronavirus vaccine - weekly summary of Yellow Card reporting - GOV.UK (www.gov.uk) Updated 11th November 2021

7) Information for healthcare professionals on myocarditis and pericarditis following COVID-19 vaccination - GOV.UK (www.gov.uk)

8) Joint Committee on Vaccination and Immunisation (JCVI) advice on COVID-19 vaccination in people aged 16 to 17 years: 15 November 2021 - GOV.UK (www.gov.uk)

9) Emergency Care Data Set (ECDS) - NHS Digital

10) SNOMED CT - NHS Digital

11) Denominators for COVID-19 vaccination statistics Available at https://www.england.nhs.uk/statistics/wp-content/uploads/sites/2/2021/05/Denominators-for-COVID-19-vaccination-statistics.docx. Accessed 31st August 2021

12) Whitaker HJ, Farrington CP, Spiessens B, Musonda P. Tutorial in biostatistics: the self-controlled case series method. Stat Med. 2006 May 30;25(10):1768-97. doi: 10.1002/sim.2302.
13) COVID-19 Greenbook chapter 14a (publishing.service.gov.uk)

14)
https://www.bing.com/search?q=NIMS+ethnicity+grouping&cvid=bc0ff142399642a0bc7cbeaa190b043c&aqs=edge.0.69i59l3j0j69i57j0l2.7221j0j9&FORM=ANAB01&PC=U531

15) COVID-19 Vaccine (Temporary Authorisation – Review Product Information tab before reading) - Summary of Product Characteristics (SmPC) - (emc) (medicines.org.uk)

16) Summary of Product Characteristics for Spikevax - GOV.UK (www.gov.uk)

17) Farrington CP, Whitaker HJ, Hocine MN. Case series analysis for censored, perturbed, or curtailed post-event exposures. Biostatistics 2009;10:3–16, http://dx.doi.org/10.1093/biostatistics/kxn013.

18) Stowe J, Andrews N, Ladhani S, Miller E. The risk of intussusception following monovalent rotavirus vaccination in England: A self-controlled case-series evaluation. Vaccine. 2016 Jul 12;34(32):3684-9. doi: 10.1016/j.vaccine.2016.04.050. Epub 2016 Jun 7.

19) Patone et al. Risks of myocarditis, pericarditis, and cardiac arrhythmias associated with COVID-19 vaccination or SARS-CoV-2 infection. Nature Medicine. 2022; 28: 410-422. DOI: 10.1038/s41591-021-01630-0.
