# Supplementary material for: Risk of myocarditis and pericarditis after a COVID-19 mRNA vaccine booster and after COVID-19 in those with and without prior SARS-CoV-2 infection: A self-controlled case series analysis in England
Source: PLoS Med. 2023 Jun 7;20(6):e1004245. doi: 10.1371/journal.pmed.1004245 (PMC10286992; doi:10.1371/journal.pmed.1004245)
Supplement: S1 Appendix — Checking the self-controlled case series assumption of no long-term event-dependence. Figure A. Plots of preexposure and postexposure relative incidence, by preexposure period length, SUS data to 6 February 2022. Figure B. Plots of preexposure and postexposure relative incidence, by preexposure period length, ECDS data to 6 February 2022. Table A. Relative incidence estimates from the standard SCCS model and the event-dependent exposures SCCS model, SUS first cases in individuals with no recorded positive SARS-CoV-2 test before the end of the observation period (6 February 2022), N = 1,977. Table B. Relative incidence estimates from the standard SCCS model and the event-dependent exposures SCCS model, ECDS first cases in individuals with no recorded positive SARS-CoV-2 test before the end of the observation period (6 February 2022), N = 3,553. (DOCX) [file pmed.1004245.s003.docx]

**Supplementary material**

**S1. Checking the self-controlled case series assumption of no long-term event-dependence**

Although a second dose of vaccine is not contraindicated if an episode of myocarditis or pericarditis occurs after the first dose for the BNT162B2 and mRNA-1273 vaccines myocarditis or pericarditis is listed under Section 4.4 Special Warnings and Precautions for Use, so those with an event after a first or second dose may be advised not to get another dose. Due to the SCCS method’s assumption that the vaccination must be independent of the occurrence of the event the main analysis accounted for short-term dependence by inclusion of a 21-day pre-vaccination window, but longer-term dependence was investigated in two ways.

First, the length of the pre-exposure period was varied from 7 to 56 days in 7-day increments, and a model with no pre-exposure period was also fitted. The pre-vaccination and post-vaccination relative incidences were then plotted against the length of the pre-exposure window [1]. If the relative incidence 0-13 days post exposure plateaus with increasing length of pre-exposure interval, this will indicate that event dependence is short-term.

Figures A and B give plots of the relative incidence within the pre-vaccination period and for post-vaccination period, for varying lengths of the pre-vaccination period for the SUS and ECDS datasets, respectively. As the pre-exposure period increased from 0 to 21 days, the pre-vaccination RI increased while the post-vaccination RI dropped a little, indicating some short-term dependence. However, overall conclusions were unaltered regardless of the pre-exposure period length. Analyses suggest that the 21-day pre-exposure window length is appropriate.

Second, long term dependence was investigated by comparing results of the standard SCCS model with the event-dependent exposures SCCS model [1,2]. To avoid the additional complication of allowing for SARS-CoV-2 infection exposures, this analysis was restricted to those individuals with no recorded positive test.

Tables A and B give the relative incidence for the standard and event-dependent models, for the SUS and ECDS datasets, respectively. The relative incidence varied a little between models, and the mRNA dose 1 and dose 2 effects were generally a little higher when estimated using the standard SCCS model than the event-dependent model, but overall conclusions were unaltered.

Overall, these sensitivity analyses gave us no reason for major concern regarding the validity of our main SCCS analysis. The addition of the cohort study, which should be unaffected by event-dependent exposures, will further ensure that our conclusions are robust.

References

1. Farrington CP, Whitaker HJ and Ghebremichael-Weldeselassie Y. Self-controlled case series studies: a modelling guide with R. Chapman and Hall / CRC press. 2018.
2. Farrington CP, Whitaker HJ and Hocine MN. Case series analysis for censored, perturbed or curtailed post-event exposures. Biostatistics, 2009, 10(1): 3-16.

Figure A Plots of pre-exposure and post-exposure relative incidence, by pre-exposure period length, SUS data to 6^th^ Feb 2022.


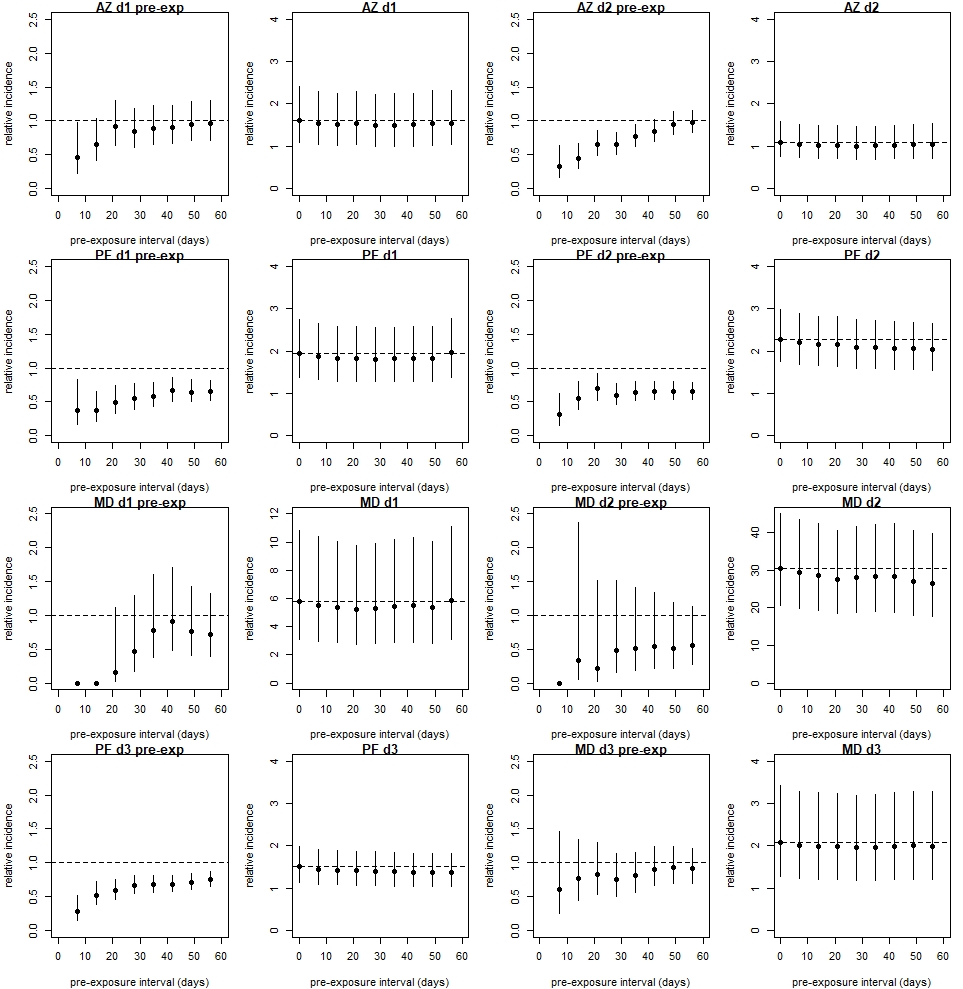


AZ = ChAdOx1, PF = BNT162b2, MD = mRNA-1273, d1 = dose 1, d2 = dose 2, d3 = dose 3

Figure B . Plots of pre-exposure and post-exposure relative incidence, by pre-exposure period length, ECDS data to 6^th^ Feb 2022.
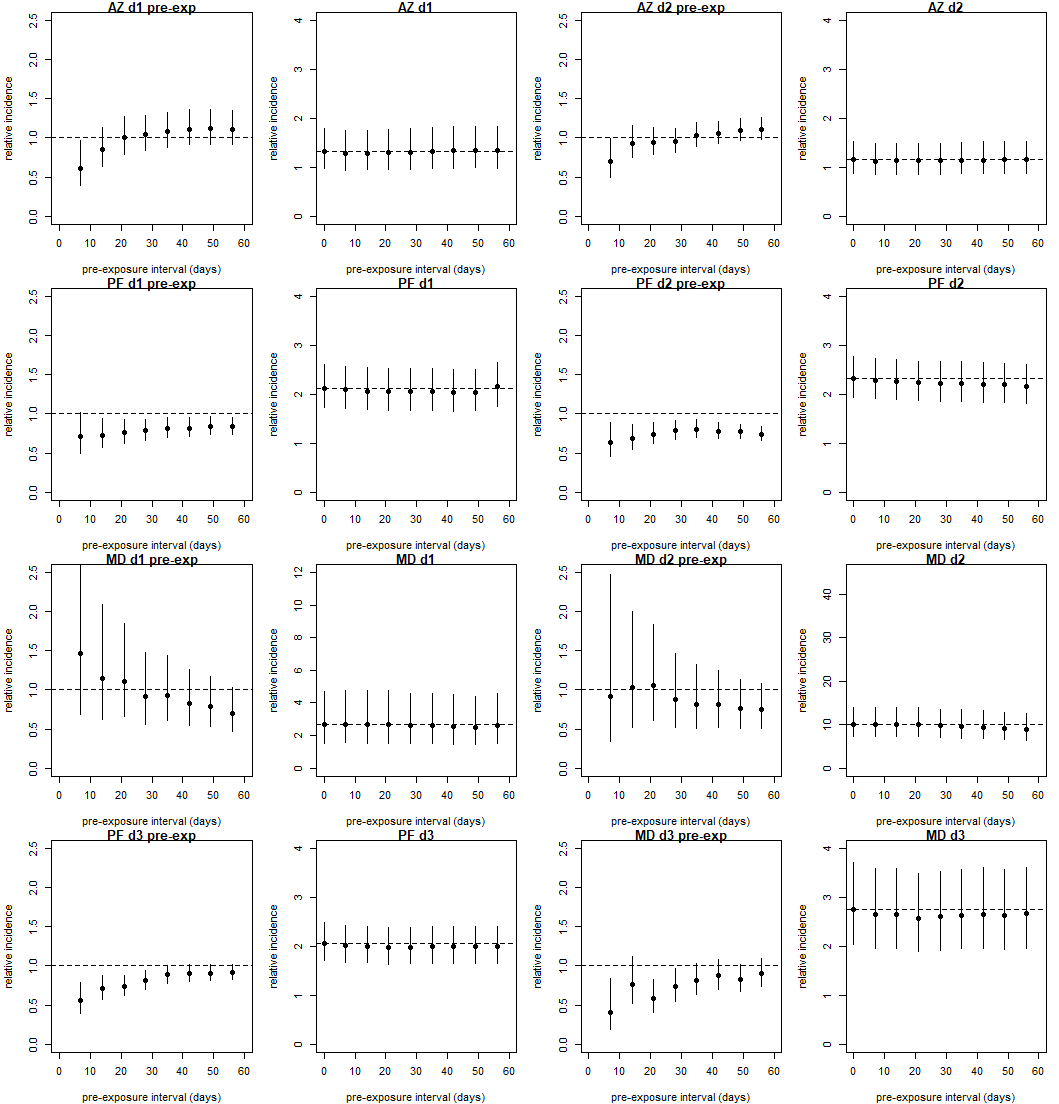


AZ = ChAdOx1, PF = BNT162b2, MD = mRNA-1273, d1 = dose 1, d2 = dose 2, d3 = dose 3

Table A : relative incidence estimates from the standard SCCS model and the event-dependent exposures SCCS model, SUS first cases in individuals with no recorded positive SARS-CoV-2 test before the end of the observation period (6^th^ Feb 2022), N = 1977

|  |  | standard SCCS | | event-dependent exposures SCCS | |
| --- | --- | --- | --- | --- | --- |
|  |  | **RI (95% CI)** | **p-value** | **RI (95% CI)** | **p-value** |
| ChAdOx1 | pre dose 1: -21 - -1d | 0.96 (0.6, 1.55) | 0.872 |  |  |
|  | dose 1: 0-6d | 1.49 (0.86, 2.58) | 0.147 | 1.54 (0.9, 2.65) | 0.116 |
|  | dose 1: 7-13d | 1.12 (0.62, 2.01) | 0.697 | 1.14 (0.64, 2.05) | 0.651 |
|  | pre dose 2: -21 - -1d | 0.59 (0.4, 0.85)** | 0.005 |  |  |
|  | dose 2: 0-6d | 1.2 (0.77, 1.87) | 0.409 | 1.26 (0.81, 1.96) | 0.304 |
|  | dose 2: 7-13d | 1.09 (0.68, 1.73) | 0.723 | 1.13 (0.71, 1.8) | 0.606 |
| BNT162b2 | pre dose 1: -21 - -1d | 0.51 (0.29, 0.91) | 0.024 |  |  |
|  | dose 1: 0-6d | 2.32 (1.49, 3.6)*** | <0.001 | 1.93 (1.23, 3.03)** | 0.004 |
|  | dose 1: 7-13d | 1.1 (0.61, 2.02) | 0.757 | 0.95 (0.52, 1.73) | 0.856 |
|  | pre dose 2: -21 - -1d | 0.86 (0.62, 1.19) | 0.418 |  |  |
|  | dose2: 0-6d | 2.21 (1.57, 3.13)*** | <0.001 | 1.84 (1.29, 2.64)*** | <0.001 |
|  | dose 2: 7-13d | 1.2 (0.76, 1.89) | 0.437 | 1.06 (0.67, 1.69) | 0.798 |
|  | pre dose 3: -21 - -1d | 0.54 (0.39, 0.75)*** | <0.001 |  |  |
|  | dose 3: 0-6d | 1.56 (1.12, 2.17)** | 0.009 | 1.67 (1.18, 2.34)** | 0.003 |
|  | dose 3: 7-13d | 1.43 (1.01, 2.03) | 0.046 | 1.51 (1.05, 2.17) | 0.027 |
| mRNA-1273 | pre dose 1: -21 - -1d | 0.31 (0.04, 2.27) | 0.251 |  |  |
|  | dose 1: 0-6d | 7.54 (3.54, 16.05)*** | <0.001 | 6.84 (3.2, 14.62)*** | <0.001 |
|  | dose 1: 7-13d | 1.9 (0.46, 7.82) | 0.376 | 1.71 (0.41, 7.17) | 0.46 |
|  | pre dose 2: -21 - -1d | 0.43 (0.06, 3.14) | 0.405 |  |  |
|  | dose2: 0-6d | 27.9 (16.13, 48.27)*** | <0.001 | 19.59 (10.77, 35.65)*** | <0.001 |
|  | dose 2: 7-13d | 2.5 (0.6, 10.45) | 0.21 | 1.78 (0.41, 7.66) | 0.438 |
|  | pre dose 3: -21 - -1d | 0.69 (0.38, 1.28) | 0.242 |  |  |
|  | dose 3: 0-6d | 2.29 (1.27, 4.14)** | 0.006 | 2.38 (1.21, 4.72) | 0.013 |
|  | dose 3: 7-13d | 1.38 (0.65, 2.95) | 0.407 | 1.42 (0.6, 3.38) | 0.423 |

** p < 0.01, *** p < 0.001

Table B : relative incidence estimates from the standard SCCS model and the event-dependent exposures SCCS model, ECDS first cases in individuals with no recorded positive SARS-CoV-2 test before the end of the observation period (6^th^ Feb 2022), N = 3553

|  |  | standard SCCS | | event-dependent exposures SCCS | |
| --- | --- | --- | --- | --- | --- |
|  |  | **RI (95% CI)** | **p-value** | **RI (95% CI)** | **p-value** |
| ChAdOx1 | pre dose 1: -21 - -1d | 0.92 (0.66, 1.29) | 0.639 |  |  |
|  | dose 1: 0-6d | 1.04 (0.65, 1.67) | 0.862 | 1.02 (0.64, 1.64) | 0.93 |
|  | dose 1: 7-13d | 1.41 (0.95, 2.08) | 0.083 | 1.38 (0.93, 2.04) | 0.103 |
|  | pre dose 2: -21 - -1d | 0.88 (0.68, 1.13) | 0.301 |  |  |
|  | dose 2: 0-6d | 0.87 (0.57, 1.32) | 0.538 | 0.88 (0.58, 1.34) | 0.575 |
|  | dose 2: 7-13d | 0.82 (0.53, 1.25) | 0.368 | 0.83 (0.54, 1.27) | 0.408 |
| BNT162b2 | pre dose 1: -21 - -1d | 0.73 (0.55, 0.98) | 0.038 |  |  |
|  | dose 1: 0-6d | 1.77 (1.31, 2.4)*** | <0.001 | 1.49 (1.09, 2.03) | 0.011 |
|  | dose 1: 7-13d | 1.08 (0.75, 1.57) | 0.672 | 0.93 (0.64, 1.36) | 0.725 |
|  | pre dose 2: -21 - -1d | 0.85 (0.68, 1.07) | 0.15 |  |  |
|  | dose2: 0-6d | 2.44 (1.93, 3.08)*** | <0.001 | 2.14 (1.68, 2.73)*** | <0.001 |
|  | dose 2: 7-13d | 1.44 (1.07, 1.95) | 0.014 | 1.29 (0.95, 1.74) | 0.087 |
|  | pre dose 3: -21 - -1d | 0.77 (0.62, 0.97) | 0.018 |  |  |
|  | dose 3: 0-6d | 1.86 (1.44, 2.39)*** | <0.001 | 1.91 (1.45, 2.5)*** | <0.001 |
|  | dose 3: 7-13d | 1.46 (1.1, 1.94)** | 0.008 | 1.51 (1.11, 2.04)** | 0.008 |
| mRNA-1273 | pre dose 1: -21 - -1d | 1 (0.49, 2.06) | 0.988 |  |  |
|  | dose 1: 0-6d | 1.47 (0.54, 4) | 0.457 | 1.22 (0.44, 3.41) | 0.705 |
|  | dose 1: 7-13d | 2.19 (0.96, 4.98) | 0.065 | 1.82 (0.77, 4.28) | 0.171 |
|  | pre dose 2: -21 - -1d | 1.14 (0.55, 2.36) | 0.994 |  |  |
|  | dose2: 0-6d | 9.9 (6.25, 15.68)*** | <0.001 | 8.42 (5.1, 13.9)*** | <0.001 |
|  | dose 2: 7-13d | 0.43 (0.06, 3.08) | 0.394 | 0.37 (0.05, 2.62) | 0.317 |
|  | pre dose 3: -21 - -1d | 0.58 (0.35, 0.94) | 0.018 |  |  |
|  | dose 3: 0-6d | 2.2 (1.41, 3.44)*** | <0.001 | 2.24 (1.35, 3.72)** | 0.002 |
|  | dose 3: 7-13d | 1.7 (1.02, 2.81) | 0.04 | 1.8 (1.03, 3.14) | 0.038 |

** p < 0.01, *** p < 0.001
