# Supplementary material for: Risk of myocarditis and pericarditis after a COVID-19 mRNA vaccine booster and after COVID-19 in those with and without prior SARS-CoV-2 infection: A self-controlled case series analysis in England
Source: PLoS Med. 2023 Jun 7;20(6):e1004245. doi: 10.1371/journal.pmed.1004245 (PMC10286992; doi:10.1371/journal.pmed.1004245)
Supplement: S2 Appendix — Table A. Adjusted (adjusted for time period (4 weekly period)) RI of admissions with myocarditis after a COVID-19 vaccine by postvaccination risk interval in 16–24 and 25–39 year olds and 16–39 year olds by gender in SUS using the SCCS analysis—whole study period to 6 February 2022. Table B. Adjusted (for time period (4 weekly period)) RI of admissions with pericarditis after a COVID-19 vaccine by postvaccination risk interval by age group in SUS using the SCCS analysis—whole study period to 6 February 2022. Table C. Adjusted (for time period (4 weekly period)) RI of attendances with myocarditis or pericarditis after a COVID-19 vaccine by postvaccination risk interval by age group in ECDS using the SCCS analysis—whole study period to 6 February 22. Table D. Adjusted (for time period (4 weekly period)) RI of admissions with myocarditis or pericarditis in SUS using the SCCS analysis in risk periods after a COVID-19 vaccine or a SARS-CoV-2 infection with booster doses stratified by vaccine given for priming. Table E. Adjusted (for time period (4 weekly period)) relative risk (aRR) of attendances with myocarditis or pericarditis in SUS using a cohort analysis after a COVID-19 vaccine by postvaccination risk interval. Adjusted for time period, age group, gender, region, ethnic group, CEV, and other clinical risk group. Table F. Adjusted (for time period (4 weekly period)) RI of admissions with myocarditis or pericarditis after a COVID-19 vaccine by postvaccination risk interval in SUS using the SCCS analysis with data up to 23 August 2021. Table G. Adjusted (for time period (4 weekly period)) RI of hospital admission in SUS with myocarditis or pericarditis after a COVID-19 vaccine by postvaccination risk interval in 16–39 year olds with or without a prior SARS-CoV-2 infection using the SCCS analysis—whole study period to 6 February 22. Table H. Relative incidence (adjusted for time period (4 weekly period)) using the SCCS analysis and aRR of attendances in 12–15 year old [file pmed.1004245.s004.docx]

**S2 Appendix**

Table A: Adjusted (adjusted for time period (4 weekly period)) relative incidence (RI) of admissions with Myocarditis after a COVID,19 vaccine by post-vaccination risk interval in 16-24 and 25-39 year olds and 16-39 year olds by gender in SUS using the SCCS analysis, whole study period to 6th Feb 2022

|  |  |  | **ages 16-24, n = 335** | | | | | **ages 25-39, n = 430** | | | |  |
| --- | --- | --- | --- | --- | --- | --- | --- | --- | --- | --- | --- | --- |
| **vaccination status** | | **Interval (days)** | **case count** | | **person years** | **RI (95% CI)** | **p value** | **case count** | **person years** | **RI (95% CI)** | **p value** | |
| **Baseline** | | | | 201 | 245 |  |  | 285 | 299.1 |  |  | |
| **ChAdOx1,S** | **Dose 1** | 0 to 6 | 5 | | 0.6 | 24.48  (9.33, 64.27) | <0.001 | 4 | 1.7 | 2.07  (0.68, 6.31) | 0.203 | |
|  |  | 7 to 13 | n<2 | | 0.6 |  |  | n<2 | 1.8 |  |  | |
|  | **Dose 2** | 0 to 6 | 2 | | 0.6 | 7.73  (1.83, 32.68) | 0.005 | n<2 | 2 |  |  | |
|  |  | 7 to 13 | n<2 | | 0.6 |  |  | 6 | 2 | 2.92  (1.24, 6.85) | 0.014 | |
| **BNT162b2** | **Dose 1** | 0 to 6 | 9 | | 4.4 | 2.19  (1.12, 4.28) | 0.021 | 9 | 4.6 | 2.02  (1.01, 4.03) | 0.046 | |
|  |  | 7 to 13 | 6 | | 4.5 | 1.39  (0.62, 3.14) | 0.423 | 8 | 4.8 | 1.66  (0.81, 3.43) | 0.169 | |
|  | **Dose 2** | 0 to 6 | 22 | | 3.5 | 5.93  (3.78, 9.29) | <0.001 | 18 | 4.4 | 4.28  (2.6, 7.05) | <0.001 | |
|  |  | 7 to 13 | 9 | | 3.5 | 2.46  (1.25, 4.82) | 0.009 | 8 | 4.4 | 1.91  (0.93, 3.91) | 0.077 | |
|  | **Booster** | 0 to 6 | 11 | | 1.1 | 11.21  (5.78, 21.73) | <0.001 | 6 | 2.4 | 2.12  (0.91, 4.94) | 0.081 | |
|  |  | 7 to 13 | 4 | | 1 | 4.6  (1.66, 12.77) | 0.003 | 2 | 2.3 | 0.75  (0.18, 3.09) | 0.693 | |
| **mRNA-1273** | **Dose 1** | 0 to 6 | 7 | | 0.9 | 21.74  (9.42, 50.19) | <0.001 | n<2 | 0.8 |  |  | |
|  |  | 7 to 13 | n<2 | | 0.9 |  |  | n<2 | 0.8 |  |  | |
|  | **Dose 2** | 0 to 6 | 24 | | 0.7 | 102.3 ‘(49.88, 209.6) | <0.001 | 14 | 0.7 | 29.67  (14.58, 60.4) | <0.001 | |
|  |  | 7 to 13 | n<2 | | 0.7 |  |  | n<2 | 0.7 |  |  | |
|  | **Booster** | 0 to 6 | 7 | | 0.4 | 24.76 (9.69, 63.31) | <0.001 | 4 | 1 | 3.5 (1.22, 10.02) | 0.02 | |
|  |  | 7 to 13 | n<2 | | 0.4 |  |  | n<2 | 1 |  |  | |
| COVID infection test day 0 | |  | 7 | | 0.5 | 12.66 (5.78, 27.73) | <0.001 | 3 | 0.6 | 4.54  (1.44, 14.29) | 0.01 | |
| post COVID infection:  1-27d | |  | 15 | | 11.6 | 1.2 (0.69, 2.08) | 0.526 | 34 | 17 | 2.01 (1.37, 2.94) | <0.001 | |
|  |  |  | **ages 16-39 and male, n = 577** | | | | | **ages 16-39 and female, n = 188** | | | |  |
| **vaccination status** | | **Interval (days)** | **case count** | | **person years** | **RI (95% CI)** | **p value** | **case count** | **person years** | **RI (95% CI)** | **p value** | |
| **Baseline** | | | | 348 | 410.6 |  |  | 138 | 133.4 |  |  | |
| **ChAdOx1-S** | **Dose 1** | 0 to 6 | 6 | | 1.6 | 6.06  (2.52, 14.55) | <0.001 | 3 | 0.6 | 3.46 (0.82, 14.53) | 0.09 | |
|  |  | 7 to 13 | n<2 | | 1.7 |  |  | n<2 | 0.7 |  |  | |
|  | **Dose 2** | 0 to 6 | 2 | | 1.8 | 1.58  (0.38, 6.53) | 0.531 | n<2 | 0.8 |  |  | |
|  |  | 7 to 13 | 6 | | 1.8 | 4.57  (1.94, 10.77) | <0.001 | n<2 | 0.8 |  |  | |
| **BNT162b2** | **Dose 1** | 0 to 6 | 14 | | 7 | 2.38  (1.37, 4.13) | 0.002 | 4 | 2 | 1.9 (0.7, 5.17) | 0.209 | |
|  |  | 7 to 13 | 10 | | 7.1 | 1.6  (0.84, 3.05) | 0.152 | 4 | 2.1 | 1.76 (0.65, 4.79) | 0.268 | |
|  | **Dose 2** | 0 to 6 | 34 | | 6.1 | 5.79  (3.99, 8.4) | <0.001 | 6 | 1.9 | 3.35 (1.47, 7.68) | 0.004 | |
|  |  | 7 to 13 | 15 | | 6 | 2.54  (1.49, 4.32) | <0.001 | 2 | 1.9 | 1.12 (0.28, 4.56) | 0.872 | |
|  | **Booster** | 0 to 6 | 11 | | 2.4 | 4.5  (2.36, 8.6) | <0.001 | 6 | 1.1 | 4.06  (1.74, 9.47) | 0.001 | |
|  |  | 7 to 13 | 6 | | 2.3 | 2.55  (1.09, 5.93) | 0.03 | n<2 | 1.1 |  |  | |
| **mRNA-1273** | **Dose 1** | 0 to 6 | 7 | | 1.5 | 10.42  (4.5, 24.12) | <0.001 | n<2 | 0.2 |  |  | |
|  |  | 7 to 13 | n<2 | | 1.5 |  |  | n<2 | 0.2 |  |  | |
|  | **Dose 2** | 0 to 6 | 35 | | 1.2 | 60.26  (34.9, 104.04) | <0.001 | 3 | 0.1 | 32.78  (7.32, 146.9) | <0.001 | |
|  |  | 7 to 13 | n<2 | | 1.2 |  |  | n<2 | 0.1 |  |  | |
|  | **Booster** | 0 to 6 | 11 | | 1.1 | 10.5  (5.23, 21.06) | <0.001 | n<2 | 0.2 |  |  | |
|  |  | 7 to 13 | n<2 | | 1.1 |  |  | n<2 | 0.2 |  |  | |
| COVID infection test day 0 | |  | 9 | | 0.8 | 10.22  (5.17, 20.19) | <0.001 | n<2 | 0.3 |  |  | |
| post COVID infection:  1 -27d | |  | 38 | | 21.1 | 1.8 (1.26, 2.59) | 0.001 | 11 | 7.5 | 1.3 (0.68, 2.47) | 0.428 | |

(AZ = ChAdOx1, PF= BNT126b2, MD=mRNA-1273)

Table B: Adjusted (for time period (4 weekly period)) Relative incidence (RI) of admissions with Pericarditis after a

COVID-19 vaccine by post-vaccination risk interval by age group in SUS using the SCCS analysis, whole study period to 6th Feb 2022

|  |  |  | **ages 16-39, n = 413** | | | | **ages 40+, n = 1007** | | | |
| --- | --- | --- | --- | --- | --- | --- | --- | --- | --- | --- |
| **vaccination status** | | **Interval (days)** | **case count** | **person years** | **RI (95% CI)** | **p value** | **case count** | **person years** | **RI (95% CI)** | **p value** |
| **Baseline** | | | 308 | 291.9 |  |  | 763 | 708.1 |  |  |
| **ChAdOx1-S** | **Dose 1** | 0 to 6 | n<2 | 1.3 |  |  | 6 | 7.3 | 0.75 (0.33, 1.71) | 0.493 |
|  |  | 7 to 13 | 2 | 1.4 | 1.77 (0.42, 7.38) | 0.433 | 14 | 8.2 | 1.52 (0.88, 2.64) | 0.136 |
|  | **Dose 2** | 0 to 6 | 4 | 1.5 | 2.7 (0.96, 7.57) | 0.059 | 16 | 11.3 | 1.12 (0.68, 1.87) | 0.654 |
|  |  | 7 to 13 | 2 | 1.5 | 1.35 (0.33, 5.6) | 0.679 | 15 | 11.3 | 1.06 (0.63, 1.79) | 0.83 |
| **BNT162b2** | **Dose 1** | 0 to 6 | 9 | 4.8 | 1.88 (0.94, 3.73) | 0.073 | n<2 | 2.1 |  |  |
|  |  | 7 to 13 | 2 | 4.8 | 0.41 (0.1, 1.67) | 0.215 | 2 | 2.6 | 0.65 (0.16, 2.65) | 0.544 |
|  | **Dose 2** | 0 to 6 | 6 | 4.4 | 1.1 (0.48, 2.5) | 0.819 | 3 | 6.6 | 0.34 (0.11, 1.08) | 0.067 |
|  |  | 7 to 13 | 8 | 4.4 | 1.46 (0.71, 2.99) | 0.302 | 5 | 6.7 | 0.57 (0.23, 1.39) | 0.217 |
|  | **Booster** | 0 to 6 | n<2 | 2.3 |  |  | 11 | 12.3 | 0.8 (0.44, 1.47) | 0.471 |
|  |  | 7 to 13 | n<2 | 2.2 |  |  | 22 | 12.3 | 1.61 (1.04, 2.5) | 0.035 |
| **mRNA-1273** | **Dose 1** | 0 to 6 | n<2 | 0.8 |  |  | n<2 | 0.1 |  |  |
|  |  | 7 to 13 | 2 | 0.8 | 2.69 (0.64, 11.38) | 0.178 | n<2 | 0.1 |  |  |
|  | **Dose 2** | 0 to 6 | 4 | 0.6 | 4.82 (1.65, 14.01) | 0.004 | n<2 | 0.1 |  |  |
|  |  | 7 to 13 | n<2 | 0.6 |  |  | n<2 | 0.1 |  |  |
|  | **Booster** | 0 to 6 | n<2 | 1 |  |  | 2 | 2.3 | 0.79 (0.19, 3.23) | 0.045 |
|  |  | 7 to 13 | 2 | 1 | 1.81 (0.43, 7.65) | 0.418 | n<2 | 2.3 |  |  |
| **COVID infection test day 0** | | | n<2 | 0.6 |  |  | 7 | 0.7 | 10.52 (4.94, 22.43) | <0.001 |
| **post COVID infection: 1-27d** | | | 30 | 16.5 | 1.83 (1.22, 2.74) | 0.004 | 41 | 19.6 | 2.28 (1.62, 3.23) | <0.001 |

(AZ = ChAdOx1, PF= BNT126b2, MD=mRNA-1273)

Table C: Adjusted (for time period (4 weekly period)) Relative incidence (RI) of attendances with Myocarditis or Pericarditis after a COVID,19 vaccine by post-vaccination risk interval by age group in ECDS using the SCCS analysis, whole study period to 6th Feb 22.

| **Myocarditis** | | | | | | | | | | |
| --- | --- | --- | --- | --- | --- | --- | --- | --- | --- | --- |
|  |  |  | **ages 16-39, n = 750** | | | | **ages 40+, n = 456** | | | |
| **vaccination status** | | **Interval (days)** | **case count** | **person years** | **RI (95% CI)** | **p value** | **case count** | **person years** | **RI (95% CI)** | **p value** |
| **Baseline** | | | 476 | 532.4 |  |  | 299 | 319.1 |  |  |
| **ChAdOx1-S** | **Dose 1** | 0 to 6 | 6 | 2.2 | 4.56 (1.93, 10.76) | <0.001 | 3 | 3.8 | 0.94 (0.29, 3.01) | 0.916 |
|  |  | 7 to 13 | n<2 | 2.3 |  |  | 4 | 4.3 | 1.03 (0.37, 2.85) | 0.952 |
|  | **Dose 2** | 0 to 6 | n<2 | 2.4 |  |  | 9 | 5.4 | 1.78 (0.89, 3.54) | 0.101 |
|  |  | 7 to 13 | 3 | 2.4 | 1.7 (0.53, 5.43) | 0.369 | 5 | 5.4 | 1.01 (0.41, 2.48) | 0.991 |
| **BNT162b2** | **Dose 1** | 0 to 6 | 14 | 9.3 | 1.71 (0.99, 2.95) | 0.055 | 5 | 1.2 | 4.71 (1.82, 12.21) | 0.001 |
|  |  | 7 to 13 | 17 | 9.4 | 1.95 (1.18, 3.2) | 0.009 | n<2 | 1.5 |  |  |
|  | **Dose 2** | 0 to 6 | 27 | 8.4 | 3.32 (2.22, 4.95) | <0.001 | 4 | 2.7 | 1.37 (0.49, 3.78) | 0.546 |
|  |  | 7 to 13 | 14 | 8.3 | 1.67 (0.97, 2.87) | 0.062 | 4 | 2.7 | 1.42 (0.51, 3.91) | 0.499 |
|  | **Booster** | 0 to 6 | 21 | 3.5 | 5.46 (3.39, 8.8) | <0.001 | 18 | 5.1 | 3.18 (1.91, 5.31) | <0.001 |
|  |  | 7 to 13 | 12 | 3.4 | 3.28 (1.79, 6.01) | <0.001 | 9 | 5.1 | 1.64 (0.82, 3.27) | 0.158 |
| **mRNA-1273** | **Dose 1** | 0 to 6 | 4 | 1.4 | 5.52 (1.93, 15.77) | 0.001 | n<2 | 0.1 |  |  |
|  |  | 7 to 13 | n<2 | 1.4 |  |  | n<2 | 0.1 |  |  |
|  | **Dose 2** | 0 to 6 | 30 | 1.1 | 46.17 (26.56, 80.26) | <0.001 | n<2 | 0.1 |  |  |
|  |  | 7 to 13 | n<2 | 1.1 |  |  | n<2 | 0.1 |  |  |
|  | **Booster** | 0 to 6 | 11 | 1.5 | 6.66 (3.41, 12.99) | <0.001 | 5 | 1.2 | 3.73 (1.44, 9.65) | 0.007 |
|  |  | 7 to 13 | 3 | 1.5 | 1.77 (0.55, 5.73) | 0.341 | 3 | 1.2 | 2.02 (0.61, 6.7) | 0.248 |
| **COVID infection test day 0** | | | 8 | 1.1 | 7.05 (3.46, 14.35) | <0.001 | 9 | 0.5 | 23.34 (11.7, 46.55) | <0.001 |
| **post COVID infection: 1-27d** | | | 65 | 29.4 | 2.36 (1.77, 3.13) | <0.001 | 45 | 13 | 4.62 (3.21, 6.64) | <0.001 |
| **Pericarditis** | | | | | | | | | | |
|  |  |  | **ages 16-39, n = 2531** | | | | **ages 40+, n = 2747** | | | |
| **vaccination status** | | **Interval (days)** | **case count** | **person years** | **RI (95% CI)** | **p value** | **case count** | **person years** | **RI (95% CI)** | **p value** |
| **Baseline** | | | 1703 | 1781.4 |  |  | 2034 | 1925.4 |  |  |
| **ChAdOx1-S** | **Dose 1** | 0 to 6 | 15 | 7.9 | 2.27 (1.34, 3.84) | 0.002 | 19 | 24 | 0.78 (0.49, 1.23) | 0.286 |
|  |  | 7 to 13 | 11 | 8.4 | 1.52 (0.83, 2.8) | 0.175 | 31 | 26.2 | 1.17 (0.81, 1.69) | 0.406 |
|  | **Dose 2** | 0 to 6 | 10 | 9.6 | 1.19 (0.63, 2.25) | 0.583 | 32 | 32.6 | 0.96 (0.67, 1.37) | 0.809 |
|  |  | 7 to 13 | 10 | 9.6 | 1.18 (0.63, 2.23) | 0.599 | 29 | 32.7 | 0.86 (0.59, 1.25) | 0.426 |
| **BNT162b2** | **Dose 1** | 0 to 6 | 51 | 30.6 | 1.8 (1.35, 2.4) | <0.001 | 6 | 6.7 | 0.85 (0.38, 1.9) | 0.684 |
|  |  | 7 to 13 | 38 | 30.9 | 1.31 (0.94, 1.82) | 0.107 | 3 | 8.3 | 0.33 (0.11, 1.04) | 0.059 |
|  | **Dose 2** | 0 to 6 | 74 | 28.7 | 2.56 (2.02, 3.25) | <0.001 | 15 | 16.6 | 0.86 (0.51, 1.44) | 0.558 |
|  |  | 7 to 13 | 42 | 28.5 | 1.45 (1.06, 1.98) | 0.019 | 24 | 16.5 | 1.37 (0.91, 2.07) | 0.131 |
|  | **Booster** | 0 to 6 | 42 | 14.9 | 2.8 (2.03, 3.86) | <0.001 | 39 | 32.2 | 1.08 (0.78, 1.5) | 0.632 |
|  |  | 7 to 13 | 18 | 14.6 | 1.23 (0.76, 1.97) | 0.398 | 45 | 32 | 1.26 (0.93, 1.71) | 0.13 |
| **mRNA-1273** | **Dose 1** | 0 to 6 | 8 | 3.9 | 2.44 (1.19, 4.99) | 0.015 | n<2 | 0.8 |  |  |
|  |  | 7 to 13 | 13 | 3.9 | 3.85 (2.17, 6.82) | <0.001 | n<2 | 0.8 |  |  |
|  | **Dose 2** | 0 to 6 | 11 | 3.3 | 3.52 (1.9, 6.55) | <0.001 | 2 | 0.7 | 2.89 (0.69, 12.11) | 0.148 |
|  |  | 7 to 13 | 5 | 3.3 | 1.6 (0.65, 3.93) | 0.301 | n<2 | 0.7 |  |  |
|  | **Booster** | 0 to 6 | 20 | 5.9 | 3.48 (2.19, 5.55) | <0.001 | 10 | 8.5 | 1.1 (0.58, 2.07) | 0.775 |
|  |  | 7 to 13 | 12 | 5.9 | 2.06 (1.15, 3.71) | 0.016 | 13 | 8.5 | 1.43 (0.82, 2.51) | 0.208 |
| **COVID infection test day 0** | | | 19 | 3.7 | 5.11 (3.24, 8.07) | <0.001 | 18 | 2.6 | 7.03 (4.4, 11.25) | <0.001 |
| **post COVID infection: 1-27d** | | | 157 | 96.8 | 1.66 (1.4, 1.98) | <0.001 | 125 | 67.9 | 1.89 (1.56, 2.3) | <0.001 |

(AZ = ChAdOx1, PF= BNT126b2, MD=mRNA-1273)

Table D: Adjusted (for time period (4 weekly period)) relative incidence (RI) of admissions with Myocarditis or Pericarditis in SUS using the SCCS analysis in risk periods after a COVID-19 vaccine or a SARS-CoV-2 infection with booster doses stratified by vaccine given for priming

|  |  |  | **ages 16,39, n = 1172** | | | | **ages 40+, n = 2140** | | | | |
| --- | --- | --- | --- | --- | --- | --- | --- | --- | --- | --- | --- |
| **vaccination status** | | **Interval (days)** | **case count** | **person years** | **RI (95% CI)** | **p value** | **case count** | **person years** | **RI (95% CI)** | **p value** |  |
| **Baseline** | | | 789 | 831.8 |  |  | 1586 | 1508.8 |  |  | |
| **ChAdOx1-S (AZ)** | **Dose 1** | 0 to 6 | 9 | 3.5 | 3.19 (1.57, 6.45) | 0.001 | 17 | 15.1 | 1.1 (0.67, 1.81) | 0.700 |  |
|  |  | 7 to 13 | 3 | 3.7 | 1.08 (0.34, 3.42) | 0.897 | 23 | 17.1 | 1.26 (0.82, 1.93) | 0.295 |  |
|  | **Dose 2** | 0 to 6 | 6 | 4.1 | 1.7 (0.74, 3.87) | 0.209 | 23 | 23.8 | 0.84 (0.55, 1.28) | 0.414 |  |
|  |  | 7 to 13 | 8 | 4.1 | 2.21 (1.07, 4.53) | 0.031 | 22 | 23.8 | 0.81 (0.53, 1.24) | 0.331 |  |
| **BNT162b2 (PF)** | **Dose 1** | 0 to 6 | 27 | 13.7 | 2.12 (1.42, 3.15) | <0.001 | 6 | 4.4 | 1.17 (0.5, 2.72) | 0.719 |  |
|  |  | 7 to 13 | 16 | 13.9 | 1.19 (0.72, 1.98) | 0.493 | 5 | 5.4 | 0.82 (0.33, 2.02) | 0.670 |  |
|  | **Dose 2** | 0 to 6 | 46 | 12.3 | 3.68 (2.7, 5.01) | <0.001 | 9 | 13.9 | 0.56 (0.29, 1.08) | 0.083 |  |
|  |  | 7 to 13 | 24 | 12.2 | 1.9 (1.25, 2.87) | 0.002 | 10 | 13.8 | 0.62 (0.33, 1.16) | 0.134 |  |
|  | **Booster post AZ priming** | 0 to 6 | 4 | 1.8 | 1.87 (0.68, 5.14) | 0.228 | 19 | 14.7 | 1.13 (0.71, 1.8) | 0.595 |  |
|  |  | 7 to 13 | 2 | 1.7 | 1.01 (0.25, 4.12) | 0.991 | 25 | 14.6 | 1.49 (0.99, 2.24) | 0.056 |  |
|  | **Booster post PF priming** | 0 to 6 | 14 | 3.7 | 3.23 (1.85, 5.64) | <0.001 | 14 | 10.7 | 1.2 (0.7, 2.06) | 0.503 |  |
|  |  | 7 to 13 | 5 | 3.5 | 1.27 (0.52, 3.11) | 0.607 | 21 | 10.7 | 1.8 (1.15, 2.81) | 0.010 |  |
|  | **Booster post MD priming** | 0 to 6 | n<2 | 0.3 |  |  | n<2 | 0.1 |  |  |  |
|  |  | 7 to 13 | n<2 | 0.3 |  |  | n<2 | 0.1 |  |  |  |
| **mRNA-1273 (MD)** | **Dose 1** | 0 to 6 | 9 | 2.5 | 5.53 (2.73, 11.16) | <0.001 | 2 | 0.4 | 4.42 (1.01, 19.32) | 0.048 |  |
|  |  | 7 to 13 | 3 | 2.5 | 1.84 (0.58, 5.88) | 0.302 | n<2 | 0.4 |  |  |  |
|  | **Dose 2** | 0 to 6 | 42 | 2 | 29.82 (19.52, 45.55) | <0.001 | n<2 | 0.3 |  |  |  |
|  |  | 7 to 13 | 2 | 2 | 1.4 (0.34, 5.76) | 0.645 | n<2 | 0.3 |  |  |  |
|  | **Booster post AZ priming** | 0 to 6 | 2 | 0.7 | 2.45 (0.58, 10.33) | 0.222 | 5 | 3.9 | 1.1 (0.45, 2.71) | 0.828 |  |
|  |  | 7 to 13 | n<2 | 0.7 |  |  | 4 | 3.9 | 0.9 (0.33, 2.44) | 0.835 |  |
|  | **Booster post PF priming** | 0 to 6 | 9 | 1.3 | 6.06 (2.95, 12.47) | <0.001 | n<2 | 1.1 |  |  |  |
|  |  | 7 to 13 | 2 | 1.3 | 1.47 (0.36, 6.09) | 0.593 | n<2 | 1.1 |  |  |  |
|  | **Booster post MD priming** | 0 to 6 | n<2 | 0.3 |  |  | n<2 | 0.1 |  |  |  |
|  |  | 7 to 13 | n<2 | 0.3 |  |  | n<2 | 0.1 |  |  |  |
| **COVID infection test day 0** | | | 11 | 1.7 | 5.95 (3.26, 10.88) | <0.001 | 27 | 1.6 | 18.63 (12.58, 27.6) | <0.001 |  |
| **post COVID infection: 1-27d** | | | 79 | 44.8 | 1.73 (1.35, 2.22) | <0.001 | 112 | 43.5 | 2.96 (2.39, 3.68) | <0.001 |  |

(AZ = ChAdOx1, PF= BNT126b2, MD=mRNA-1273)

Table E: Adjusted (for time period (4 weekly period)) Relative Risk (aRR) of attendances with Myocarditis or Pericarditis in SUS using a cohort analysis after a COVID-19 vaccine by post-vaccination risk interval. (AZ = ChAdOx1-S, PF= BNT126b2, MD=mRNA-1273) Adjusted for time period, age group gender, region, ethnic group CEV and other clinical risk group.

|  |  |  | **SUS: 16-39** | | | | | | | **SUS: 40+** | | | | | |
| --- | --- | --- | --- | --- | --- | --- | --- | --- | --- | --- | --- | --- | --- | --- | --- |
|  | **vaccination status** | **Interval (days)** | **case count** | **person years** | **rate per 100,000 pyrs** | **Crude RR** | **aRR with 95%CI** | **p value** | | **case count** | **person years** | **rate per 100,000 pyrs** | **Crude RR** | **aRR with 95%CI** | **p value** |
|  | **unvaccinated** |  | 580 | 9667615 | 6.00 | baseline | baseline |  | | 301 | 4736185 | 6.36 | baseline | baseline |  |
| **Primary course ChAdOx1-S** | **Dose 1** | 0 to 6 | 9 | 40266 | 22.35 | 3.73 | 3.03  (1.54,5.96) | | 0.001 | 17 | 227825 | 7.46 | 1.17 | 1.28 (0.78,2.12) | 0.318 |
|  |  | 7 to 13 | 4 | 42819 | 9.34 | 1.56 | 1.26  (0.46,3.42) | 0.638 | | 23 | 252004 | 9.13 | 1.44 | 1.47 (0.95,2.27) | 0.080 |
|  |  | 14+ | 42 | 502792 | 8.35 | 1.39 | 1.04  (0.75,1.44) | 0.797 | | 275 | 3020709 | 9.10 | 1.43 | 1.22 (1.02,1.46) | 0.023 |
|  | **Dose 2** | 0 to 6 | 6 | 47768 | 12.56 | 2.09 | 1.56  (0.69,3.52) | 0.282 | | 23 | 335998 | 6.85 | 1.08 | 0.88 (0.57,1.35) | 0.569 |
|  |  | 7 to 13 | 8 | 47767 | 16.75 | 2.79 | 2.03  (1,4.13) | 0.049 | | 22 | 335988 | 6.55 | 1.03 | 0.83 (0.53,1.3) | 0.433 |
|  |  | 14+ | 127 | 1373090 | 9.25 | 1.54 | 0.92  (0.74,1.14) | 0.456 | | 698 | 8773113 | 7.96 | 1.25 | 0.95 (0.82,1.11) | 0.583 |
|  | **Booster BNT162b2** | 0 to 6 | 4 | 23778 | 16.82 | 2.80 | 1.59  (0.59,4.3) | 0.358 | | 19 | 222086 | 8.56 | 1.35 | 0.85 (0.53,1.38) | 0.532 |
|  |  | 7 to 13 | 2 | 23385 | 8.55 | 1.43 | 0.79  (0.19,3.21) | 0.750 | | 25 | 220987 | 11.31 | 1.78 | 1.1 (0.72,1.69) | 0.630 |
|  |  | 14+ | 13 | 157081 | 8.28 | 1.38 | 0.71  (0.4,1.25) | 0.242 | | 141 | 1970679 | 7.15 | 1.13 | 0.62 (0.48,0.79) | <0.001 |
|  | **Booster mRNA-1273** | 0 to 6 | 2 | 9601 | 20.83 | 3.47 | 2.04  (0.5,8.26) | 0.317 | | 5 | 74513 | 6.71 | 1.06 | 0.76 (0.31,1.86) | 0.552 |
|  |  | 7 to 13 | n<2 | 9582 |  |  |  |  | | 4 | 74465 | 5.37 | 0.85 | 0.6 (0.22,1.62) | 0.317 |
|  |  | 14+ | 4 | 53690 | 7.45 | 1.24 | 0.66  (0.24,1.81) | 0.427 | | 25 | 499183 | 5.01 | 0.79 | 0.54 (0.35,0.83) | 0.006 |
| **Primary course: BNT162b2** | **Dose 1** | 0 to 6 | 27 | 186142 | 14.51 | 2.42 | 2.44  (1.65,3.62) | <0.001 | | 6 | 50570 | 11.86 | 1.87 | 1.53 (0.67,3.47) | 0.303 |
|  |  | 7 to 13 | 18 | 187325 | 9.61 | 1.60 | 1.58  (0.98,2.55) | 0.055 | | 5 | 64948 | 7.70 | 1.21 | 0.96 (0.39,2.36) | 0.944 |
|  |  | 14+ | 142 | 1725699 | 8.23 | 1.37 | 1.25  (1.03,1.51) | 0.022 | | 121 | 1225180 | 9.88 | 1.55 | 1.16 (0.93,1.46) | 0.171 |
|  | **Dose 2** | 0 to 6 | 45 | 182573 | 24.65 | 4.11 | 3.9  (2.86,5.32) | <0.001 | | 9 | 162742 | 5.53 | 0.87 | 0.62 (0.31,1.21) | 0.162 |
|  |  | 7 to 13 | 23 | 181777 | 12.65 | 2.11 | 1.96  (1.28,2.98) | 0.002 | | 10 | 162873 | 6.14 | 0.97 | 0.68 (0.36,1.29) | 0.246 |
|  |  | 14+ | 298 | 3528379 | 8.45 | 1.41 | 1.18  1.01,1.38) | 0.035 | | 413 | 4527414 | 9.12 | 1.44 | 0.94 (0.79,1.11) | 0.507 |
|  | **Booster BNT162b2** | 0 to 6 | 14 | 73115 | 19.15 | 3.19 | 2.77  (1.6,4.79) | <0.001 | | 14 | 138773 | 10.09 | 1.59 | 0.95 (0.55,1.65) | 0.880 |
|  |  | 7 to 13 | 5 | 71106 | 7.03 | 1.17 | 0.98  (0.4,2.41) | 0.981 | | 21 | 138423 | 15.17 | 2.39 | 1.39 (0.88,2.21) | 0.149 |
|  |  | 14+ | 32 | 406013 | 7.88 | 1.31 | 1.05  (0.71,1.55) | 0.782 | | 151 | 1684224 | 8.97 | 1.41 | 0.72 (0.57,0.92) | 0.009 |
|  | **Booster mRNA-1273** | 0 to 6 | 8 | 27433 | 29.16 | 4.86 | 4.56  (2.22,9.35) | <0.001 | | n<2 | 13477 |  |  |  |  |
|  |  | 7 to 13 | 2 | 27343 | 7.31 | 1.22 | 1.08  (0.26,4.4) | 0.907 | | n<2 | 13466 |  |  |  |  |
|  |  | 14+ | 7 | 123213 | 5.68 | 0.95 | 0.79  (0.37,1.71) | 0.559 | | 5 | 99553 | 5.02 | 0.79 | 0.38 (0.15,0.93) | 0.035 |
| **Primary course: mRNA-1273** | **Dose 1** | 0 to 6 | 9 | 19635 | 45.84 | 7.64 | 7.46  (3.84,14.51) | <0.001 | | 2 | 5439 | 36.77 | 5.79 | 6.64 (1.65,26.73) | 0.008 |
|  |  | 7 to 13 | 3 | 19618 | 15.29 | 2.55 | 2.45  (0.78,7.67) | 0.121 | | n<2 | 5436 |  |  |  |  |
|  |  | 14+ | 22 | 166140 | 13.24 | 2.21 | 1.92 (1.24,2.96) | 0.003 | | n<2 | 46201 |  |  |  |  |
|  | **Dose 2** | 0 to 6 | 41 | 16897 | 242.65 | 40.45 | 35.36 (25.39,49.23) | <0.001 | | n<2 | 4659 |  |  |  |  |
|  |  | 7 to 13 | 2 | 16869 | 11.86 | 1.98 | 1.67 (0.41,6.74) | 0.467 | | n<2 | 4653 |  |  |  |  |
|  |  | 14+ | 29 | 301139 | 9.63 | 1.61 | 1.32 (0.9,1.93) | 0.153 | | 8 | 98988 | 8.08 | 1.27 | 1.39 (0.68,2.83) | 0.354 |
|  | **Booster BNT162b2** | 0 to 6 | n<2 | 5132 |  |  |  |  | | n<2 | 1737 |  |  |  | 0.996 |
|  |  | 7 to 13 | n<2 | 4919 |  |  |  |  | | n<2 | 1702 |  |  |  |  |
|  |  | 14+ | n<2 | 19430 |  |  |  |  | | n<2 | 8090 |  |  |  |  |
|  | **Booster mRNA-1273** | 0 to 6 | n<2 | 4088 |  |  |  |  | | n<2 | 1594 |  |  |  |  |
|  |  | 7 to 13 | n<2 | 4066 |  |  |  |  | | n<2 | 1590 |  |  |  |  |
|  |  | 14+ | n<2 | 16863 |  |  |  |  | | n<2 | 7764 |  |  |  |  |

(AZ = ChAdOx1, PF= BNT126b2, MD=mRNA-1273)

Table F: Adjusted (for time period (4 weekly period)) relative incidence (RI) of admissions with Myocarditis or Pericarditis after a

COVID-19 vaccine by post-vaccination risk interval in SUS using the SCCS analysis with data up to 23rd August 2021

|  |  |  | **ages 16-39, n = 501** | | | | **ages 40+, n = 1109** | | | |
| --- | --- | --- | --- | --- | --- | --- | --- | --- | --- | --- |
|  |  |  | **case count** | **person years** | **RI (95% CI)** | **p value** | **case count** | **person years** | **RI (95% CI)** | **p value** |
| **Baseline** | | | 338 | 424.3 |  |  | 826 | 777.9 |  |  |
| **ChAdOx1-S** | **Dose 1** | **0 to 6** | 9 | 3.5 | 2.46 (1.19, 5.08) | 0.015 | 17 | 15 | 1.02 (0.61, 1.7) | 0.793 |
|  |  | **7 to 13** | 3 | 3.7 | 0.85 (0.26, 2.72) | 0.781 | 23 | 17.1 | 1.24 (0.81, 1.91) | 0.364 |
|  | **Dose 2** | **0 to 6** | 6 | 4 | 1.36 (0.59, 3.15) | 0.472 | 23 | 23.4 | 0.88 (0.58, 1.34) | 0.512 |
|  |  | **7 to 13** | 8 | 4 | 1.77 (0.85, 3.7) | 0.127 | 22 | 23.4 | 0.84 (0.55, 1.3) | 0.415 |
| **BNT162b2** | **Dose 1** | **0 to 6** | 17 | 11.2 | 1.83 (1.1, 3.06) | 0.021 | 5 | 3.7 | 1.02 (0.4, 2.6) | 0.974 |
|  |  | **7 to 13** | 10 | 11.1 | 1.04 (0.54, 1.99) | 0.907 | 5 | 4.7 | 0.88 (0.35, 2.18) | 0.73 |
|  | **Dose 2** | **0 to 6** | 28 | 6.8 | 5.07 (3.29, 7.8) | <0.001 | 8 | 13 | 0.54 (0.27, 1.1) | 0.081 |
|  |  | **7 to 13** | 10 | 6 | 2.03 (1.05, 3.94) | 0.035 | 9 | 13 | 0.61 (0.31, 1.19) | 0.131 |
| **mRNA-1273** | **Dose 1** | **0 to 6** | 7 | 2.3 | 5 (2.13, 11.71) | <0.001 | n<2 | 0.3 |  |  |
|  |  | **7 to 13** | 2 | 2.3 | 1.43 (0.34, 6.09) | 0.628 | n<2 | 0.3 |  |  |
|  | **Dose 2** | **0 to 6** | 10 | 0.7 | 17.46 (7.15, 42.63) | <0.001 | n<2 | 0.2 |  |  |
|  |  | **7 to 13** | n<2 | 0.6 |  |  | n<2 | 0.2 |  |  |
| **COVID infection test day 0** | | | 4 | 0.5 | 8 (2.88, 22.26) | <0.001 | 7 | 0.5 | 13.58 (6.23, 29.58) | <0.001 |
| **post COVID infection:**  **1-27d** | |  | 14 | 13.5 | 1.14 (0.63, 2.04) | 0.669 | 41 | 12.7 | 2.71 (1.83, 4.01) | <0.001 |

AZ = ChAdOx1, PF= BNT126b2, MD=mRNA-1273

Table G: Adjusted (for time period (4 weekly period)) Relative incidence (RI) of hospital admission in SUS with myocarditis or pericarditis after a COVID-19 vaccine by post-vaccination risk interval in 16-39 year olds with or without a prior laboratory confirmed SARS-CoV-2 infection using the SCCS analysis , whole study period to 6th Feb 22

|  |  |  | **prior confirmed infection, n = 398** | | | | **No prior confirmed infection, n = 783** | | | |
| --- | --- | --- | --- | --- | --- | --- | --- | --- | --- | --- |
|  |  |  | **case count** | **person years** | **RI (95% CI)** | **p value** | **case count** | **person years** | **RI (95% CI)** | **p value** |
| **Baseline** | | | 240 | 224.7 |  |  | 567 | 630.1 |  |  |
| **ChAdOx1,S** | **Dose 1** | 0 to 6 | n<2 | 0.6 |  |  | 9 | 2.9 | 4.21 (2.04, 8.72) | <0.001 |
|  |  | 7 to 13 | n<2 | 0.7 |  |  | 2 | 3.1 | 0.98 (0.24, 4.02) | 0.974 |
|  | **Dose 2** | 0 to 6 | n<2 | 0.9 |  |  | 5 | 3.2 | 2.05 (0.82, 5.09) | 0.124 |
|  |  | 7 to 13 | n<2 | 0.9 |  |  | 8 | 3.2 | 3.18 (1.52, 6.63) | 0.002 |
| **BNT162b2** | **Dose 1** | 0 to 6 | 5 | 3.2 | 1.28 (0.52, 3.19) | 0.592 | 22 | 10.6 | 2.38 (1.52, 3.71) | <0.001 |
|  |  | 7 to 13 | 7 | 3.3 | 1.6 (0.74, 3.49) | 0.234 | 10 | 10.7 | 1.06 (0.56, 2.01) | 0.855 |
|  | **Dose 2** | 0 to 6 | 11 | 3.5 | 2.47 (1.32, 4.63) | 0.005 | 36 | 8.7 | 4.45 (3.12, 6.34) | <0.001 |
|  |  | 7 to 13 | 6 | 3.6 | 1.33 (0.58, 3.03) | 0.504 | 18 | 8.6 | 2.23 (1.37, 3.61) | 0.001 |
|  | **Booster** | 0 to 6 | 9 | 2.6 | 3.04 (1.45, 6.35) | 0.003 | 10 | 3.2 | 2.61 (1.35, 5.04) | 0.004 |
|  |  | 7 to 13 | 4 | 2.6 | 1.19 (0.41, 3.46) | 0.752 | 3 | 3 | 0.86 (0.27, 2.73) | 0.801 |
| **mRNA-1273** | **Dose 1** | 0 to 6 | 2 | 0.5 | 4.91 (1.1, 21.88) | 0.037 | 7 | 2.1 | 6.07 (2.71, 13.62) | <0.001 |
|  |  | 7 to 13 | n<2 | 0.5 |  |  | 3 | 2.1 | 2.56 (0.79, 8.28) | 0.118 |
|  | **Dose 2** | 0 to 6 | 12 | 0.6 | 19.07 (8.62, 42.19) | <0.001 | 30 | 1.4 | 37.2 (22.18, 62.38) | <0.001 |
|  |  | 7 to 13 | n<2 | 0.6 |  |  | 2 | 1.4 | 2.4 (0.57, 10.09) | 0.231 |
|  | **Booster** | 0 to 6 | 4 | 1 | 4.81 (1.58, 14.64) | 0.006 | 8 | 1.3 | 5.33 (2.48, 11.43) | <0.001 |
|  |  | 7 to 13 | n<2 | 1 |  | 0.823 | 2 | 1.3 | 1.51 (0.36, 6.29) | 0.57 |
| COVID infection test day 0 | |  | 11 | 1.7 | 5.28 (2.83, 9.86) | <0.001 |  |  |  |  |
| post COVID infection:  1- 27d | |  | 79 | 44.9 | 1.51 (1.13, 2.02) | 0.005 |  |  |  |  |

AZ = ChAdOx1, PF= BNT126b2, MD=mRNA-1273

Table H: Relative Incidence (adjusted for time period (4 weekly period) using the SCCS analysis and Adjusted relative risk (aRR) of attendances in 12-15 year olds with Myocarditis or Pericarditis in ECDS after a BNT162b2 COVID-19 vaccine by post-vaccination risk interval.

| **Attendances (ECDS) ages 12-15** | | | | | | | |
| --- | --- | --- | --- | --- | --- | --- | --- |
| **Cohort analysis** | | | | | | | |
|  |  |  | **case count** | **person years** | **rate per 100,000 person years** | **aRR with 95%CI** | **p value** |
| **unvaccinated** | | | 100 | 2347285 | 4.26 | baseline |  |
| **BNT162b2** | **Dose 1** | 0 to 6 | 21 | 30029 | 69.93 | 9.61 (5.7,16.19) | < 0.001 |
|  |  | 7 to 13 | 7 | 29588 | 23.66 | 3.12 (1.4,6.97) | 0.005 |
|  |  | 14+ | 14 | 298863 | 4.68 | 0.67 (0.36,1.26) | 0.22 |
|  | **Dose 2** | 0 to 6 | 6 | 10111 | 59.34 | 7.79 (3.11,19.49) | < 0.001 |
|  |  | 7 to 13 | 2 | 8108 | 24.67 | 3.18 (0.73,13.72) | 0.121 |
|  |  | 14+ | n<2 | 17850 | 5.60 |  |  |
| **SCCS analysis** | | | | | | | |
|  | | | **case count** | **person years** |  | **RI (95% CI)** | **p value** |
| **BNT162b2** | **Baseline count** | | 47 | 69.9 |  | baseline |  |
|  | **Dose 1** | 0 to 6 | 21 | 1.6 |  | 14.43 (7.62, 27.34) | < 0.001 |
|  |  | 7 to 13 | 7 | 1.6 |  | 4.35 (1.81, 10.46) | 0.001 |
|  | **Dose 2** | 0 to 6 | 6 | 0.5 |  | 16.67 (5.39, 51.62) | < 0.001 |
|  |  | 7 to 13 | 2 | 0.4 |  | 5.72 (1.13, 29.01) | 0.035 |

AZ = ChAdOx1, PF= BNT126b2, MD=mRNA-1273
