# Supplementary material for: Risk of myocarditis and pericarditis after a COVID-19 mRNA vaccine booster and after COVID-19 in those with and without prior SARS-CoV-2 infection: A self-controlled case series analysis in England
Source: PLoS Med. 2023 Jun 7;20(6):e1004245. doi: 10.1371/journal.pmed.1004245 (PMC10286992; doi:10.1371/journal.pmed.1004245)
Supplement: Attachment — Submitted filename: Review_22-03092R1.docx [file pmed.1004245.s005.docx]

**Review of ‘Risk of myocarditis after a COVID-19 mRNA vaccine booster following homologous or heterologous priming in those with and without prior SARS-CoV-2 infection; an observational database study in England’**

The manuscript reports on the risk of hospital admission for myocarditis/pericarditis following the administration of booster doses with either of the two FDA-approved mRNA vaccines, after priming with adenovirus or mRA vaccines. This topic should be of broad appeal to the PLOS Medicine audience.

The manuscript is clearly written and the findings are important. The robustness of the study’s findings was demonstrated through consistency of the SCCS and retrospective cohort analysis results. The authors also assessed the SCCS model assumptions of no long-term event dependence. A sensitivity analysis was performed and limitations of the study were well described.

Some comments and suggestions follow.

**Major Comments**

1. The multiplicity adjustment is not well justified and appears rather arbitrary. How was the alpha-level of 0.001 selected? The multiple testing spanned time intervals, demographic subgroups, and endpoints (pericarditis, myocarditis, and myocarditis and/or pericarditis). It was not clear how many multiple tests were conducted and whether this 0.001 alpha level adequately controls the family-wise error rate (FWE) or the false-discovery rate (FDR) at a given level. I would recommend that the authors apply a method like Holm-Bonferroni to compute adjusted p-values that control the FWE at 0.05.
2. A brief section on the statistical analysis methods used should be included in the manuscript rather than simply referring to the analysis plan in the protocol.
3. Recommend providing 95%CIs on pg5 lines 74, 78
4. Appendix S2: Please explain which analyses suggest that the 21-day pre-exposure window length is appropriate.
5. Pg 12, Line 261: Good to show no overlap in 95%CIs to back up the statement.
6. Pg 15, Line 333: Add 95%CIs to AR estimates for admission.
7. Table 1: Add 95%CIs to columns with SUS and ECDS risk estimates.
8. Table 3: Add column of p-values.

**Minor Comments**

1. Pg 9, line 173: It would be helpful to explain why those who received their 3^rd^ dose before 1 Sept 2021 and those who received their 1^st^ dose before 8 Dec 2020 were excluded. What is the significance of those dates?
2. Abstract and Table 1: provide exact age range of study cohort: 12-x years. Could add a footnote to Table 1 noting the age of oldest person.
3. Appendix S2, paragraph 5, second sentence, add “generally”: mRNA dose 1 and2 effects were *generally* a little higher”
4. Figs S2A, S2B, Table S2A, S2B: Use same vaccine group labels as main manuscript. Define d1, d2 as dose 1, dose 2. Add p-values to tables.
5. Pg 10, line 210: Would it be better to call it a secondary or exploratory analysis here rather than a sensitivity analysis? It seems to be addressing a separate question than checking on the robustness of the primary analysis. Lines 211-214 do seem to appropriately describe a sensitivity analysis to check for ascertainment bias.
6. Figures 1-3 are very low resolution- quality should be improved. Please also use the same vaccine labels as main manuscript and use different colors or symbols instead of shades of grey to distinguish these.
7. Fig 3: Add 95%CIs to RI estimates.
