# Supplementary material for: Risk of myocarditis and pericarditis after a COVID-19 mRNA vaccine booster and after COVID-19 in those with and without prior SARS-CoV-2 infection: A self-controlled case series analysis in England
Source: PLoS Med. 2023 Jun 7;20(6):e1004245. doi: 10.1371/journal.pmed.1004245 (PMC10286992; doi:10.1371/journal.pmed.1004245)
Supplement: Attachment — Submitted filename: Reply_to_reviewers_final.docx [file pmed.1004245.s006.docx]

Dear Dr. Miller,

Thank you very much for submitting your manuscript "Risk of myocarditis after a COVID-19 mRNA vaccine booster following homologous or heterologous priming in those with and without prior SARS-CoV-2 infection; an observational database study in England" (PMEDICINE-D-22-03092R1) for consideration at PLOS Medicine.

Your paper was evaluated by a senior editor and discussed among all the editors here. It was also sent to independent reviewers, including a statistical reviewer. The reviews are appended at the bottom of this email and any accompanying reviewer attachments can be seen via the link below:

https://www.editorialmanager.com/pmedicine/l.asp?i=832205&l=HCLGXYKR

In light of these reviews, I am afraid that we will not be able to accept the manuscript for publication in the journal in its current form, but we would like to consider a revised version that addresses the reviewers' and editors' comments. Obviously we cannot make any decision about publication until we have seen the revised manuscript and your response, and we plan to seek re-review by one or more of the reviewers.

In revising the manuscript for further consideration, your revisions should address the specific points made by each reviewer and the editors. Please also check the guidelines for revised papers at [http://journals.plos.org/plosmedicine/s/revising-your-manuscript](http://protect-eu.mimecast.com/s/HE6iCp215ipP0gBhx33B2?domain=journals.plos.org) for any that apply to your paper. In your rebuttal letter you should indicate your response to the reviewers' and editors' comments, the changes you have made in the manuscript, and include either an excerpt of the revised text or the location (eg: page and line number) where each change can be found. Please submit a clean version of the paper as the main article file; a version with changes marked should be uploaded as a marked up manuscript.

In addition, we request that you upload any figures associated with your paper as individual TIF or EPS files with 300dpi resolution at resubmission; please read our figure guidelines for more information on our requirements: [http://journals.plos.org/plosmedicine/s/figures](http://protect-eu.mimecast.com/s/Lr6RCqZ14tJGygoFpkmVE?domain=journals.plos.org). While revising your submission, please upload your figure files to the PACE digital diagnostic tool, [https://pacev2.apexcovantage.com/](https://protect-eu.mimecast.com/s/GvvNCr914S4XRyGI3rZrS?domain=pacev2.apexcovantage.com). PACE helps ensure that figures meet PLOS requirements. To use PACE, you must first register as a user. Then, login and navigate to the UPLOAD tab, where you will find detailed instructions on how to use the tool. If you encounter any issues or have any questions when using PACE, please email us at PLOSMedicine@plos.org.

We expect to receive your revised manuscript by Feb 23 2023 11:59PM. Please email us (plosmedicine@plos.org) if you have any questions or concerns.

***Please note while forming your response, if your article is accepted, you may have the opportunity to make the peer review history publicly available. The record will include editor decision letters (with reviews) and your responses to reviewer comments. If eligible, we will contact you to opt in or out.***

We ask every co-author listed on the manuscript to fill in a contributing author statement, making sure to declare all competing interests. If any of the co-authors have not filled in the statement, we will remind them to do so when the paper is revised. If all statements are not completed in a timely fashion this could hold up the re-review process. If new competing interests are declared later in the revision process, this may also hold up the submission. Should there be a problem getting one of your co-authors to fill in a statement we will be in contact. YOU MUST NOT ADD OR REMOVE AUTHORS UNLESS YOU HAVE ALERTED THE EDITOR HANDLING THE MANUSCRIPT TO THE CHANGE AND THEY SPECIFICALLY HAVE AGREED TO IT. You can see our competing interests policy here: [http://journals.plos.org/plosmedicine/s/competing-interests](http://protect-eu.mimecast.com/s/Qv2yCvZ14tronJ3fg7bM-?domain=journals.plos.org).

Please use the following link to submit the revised manuscript:

https://www.editorialmanager.com/pmedicine/

Your article can be found in the "Submissions Needing Revision" folder.

To enhance the reproducibility of your results, we recommend that you deposit your laboratory protocols in protocols.io, where a protocol can be assigned its own identifier (DOI) such that it can be cited independently in the future. Additionally, PLOS ONE offers an option to publish peer-reviewed clinical study protocols. Read more information on sharing protocols at [https://plos.org/protocols?utm_medium=editorial-email&utm_source=authorletters&utm_campaign=protocols](https://protect-eu.mimecast.com/s/632iCwr14SgXE5WIAJGJJ?domain=plos.org)

Please ensure that the paper adheres to the PLOS Data Availability Policy (see [http://journals.plos.org/plosmedicine/s/data-availability](http://protect-eu.mimecast.com/s/9UHxCx1g4cPK5p3HN1dgM?domain=journals.plos.org)), which requires that all data underlying the study's findings be provided in a repository or as Supporting Information. For data residing with a third party, authors are required to provide instructions with contact information for obtaining the data. PLOS journals do not allow statements supported by "data not shown" or "unpublished results." For such statements, authors must provide supporting data or cite public sources that include it.

We look forward to receiving your revised manuscript.

Sincerely,

Philippa Dodd, MBBS MRCP PhD
PLOS Medicine
plosmedicine.org


-----------------------------------------------------------
Requests from the editors:

GENERAL
Please respond to all editor and reviewer comments detailed below, in full.

DATA AVAILABILITY STATEMENT
PLOS Medicine requires that the de-identified data underlying the specific results in a published article be made available, without restrictions on access, in a public repository or as Supporting Information at the time of article publication, provided it is legal and ethical to do so. Please see the policy at

[http://journals.plos.org/plosmedicine/s/data-availability](http://protect-eu.mimecast.com/s/9UHxCx1g4cPK5p3HN1dgM?domain=journals.plos.org)

and FAQs at

[http://journals.plos.org/plosmedicine/s/data-availability#loc-faqs-for-data-policy](http://protect-eu.mimecast.com/s/AIw-CyXjgiAEPGRhlPYgu?domain=journals.plos.org)

PLOS defines the “minimal data set” to consist of the data set used to reach the conclusions drawn in the manuscript with related metadata and methods, and any additional data required to replicate the reported study findings in their entirety. Authors do not need to submit their entire data set, or the raw data collected during an investigation. Please submit the following data:
The values behind the means, standard deviations and other measures reported;

The values used to build graphs;

Provided

The points extracted from images for analysis.

Provided

The Data Availability Statement (DAS) requires revision. For each data source used in your study:
a) If the data are freely or publicly available, note this and state the location of the data: within the paper, in Supporting Information files, or in a public repository (include the DOI or accession number).
b) If the data are owned by a third party but freely available upon request, please note this and state the owner of the data set and contact information for data requests (web or email address). Note that a study author cannot be the contact person for the data.
c) If the data are not freely available, please describe briefly the ethical, legal, or contractual restriction that prevents you from sharing it. Please also include an appropriate contact (web or email address) for inquiries (again, this cannot be a study author).

As previously indicated at the initial submission we are unable to place the data that was used for our study in the public domain. Below is the data availability statement that we propose to use and which has been used for other UKHSA publications using this data set and trust this is acceptable.

*The raw study data are protected and are not freely available due to data privacy laws. This work is carried out under Regulation 3 of The Health Service (Control of Patient Information) (Secretary of State for Health, 2002))(3) using patient identification information without individual patient consent. Data cannot be made publicly available for ethical and legal reasons, i.e. public availability would compromise patient confidentiality as data tables list single counts of individuals rather than aggregated data. Requests for the underlying data should be made via the UKHSA office for data release:* [*https://www.gov.uk/government/publications/accessing-ukhsa-protected-data*](https://www.gov.uk/government/publications/accessing-ukhsa-protected-data)

TITLE
Suggest “Risk of myocarditis after a COVID-19 mRNA vaccine booster following homologous or heterologous priming in those with and without prior SARS-CoV-2 infection: a self-controlled case series analysis in England” or something similar. You may wish to make further revisions in accordance with reviewer comments – please see below.

Now revised in the light of your suggestion and reviewers’ comments to:

**Risk of myocarditis after a COVID-19 mRNA vaccine booster and after COVID-19 in those with and without prior SARS-CoV-2 infection; a self-controlled case series analysis in England**

ABSTRACT
Abstract Background:
It may be helpful to expand this section further outlining why the study is important. Please ensure that the final sentence clearly states the study question.

Now expanded

Abstract methods and findings:
Line 33-34: is the word “days” missing after 1-27?

Yes this was omitted in error –now added
Please include the timeframe over which the data were collected and the main outcome measures.

Now added
Please include the number of participants (of the 50 million eligible to receive the vaccine) that were diagnosed with myocarditis and/or pericarditis (7929 individuals as per the results section of the main manuscript)

The number of hospital admissions for myocarditis or pericarditis is now added – the 7929 number is for the emergency care consultations which we do not mention in the abstract due to limited space

Please detail where data were extracted from (i.e. which databases)

Now added
Please quantify the main results with p values as well as 95% CIs. When reporting p values please report as p<0.001 (not .001) or where higher, the exact p value as p=0.002 (not .002), for example. Suggest modifying statistical reporting to read as follows: 3.23 (95% CI [1.85,5.64]; p= 0.0… or p<0.001…) and 6.06 (95% CI [2.95,12.47], p=0.00 or p<0.001)
Please include any important dependent variables that are adjusted for in the analyses.
Apart from the possibility of overestimation of the RIs, were there any other limitations of the study's methodology?

95% CIs and p values now added to all RIs and format as suggested above used when citing RIs in the text.

Abstract Conclusions:
Line 41: Please address the study implications without overreaching what can be concluded from the data; the phrase "In this study, we observed ..." may be useful. This sentence is rather long and as result not very accessible to the reader, please revise.
Line 43: suggest “…which contains less mRNA...” perhaps
Line 46: Suggest “…behind this phenomenon…” perhaps
Please interpret the study based on the results presented in the abstract, emphasizing what is new without overstating your conclusions.

Suggestions implemented

AUTHOR SUMMARY
Thank you for including an author summary. Please ensure that each bullet point is succinct, and that the language used is accessible to the general reader. It may be helpful for you to visit the journal's homepage here: [https://journals.plos.org/plosmedicine/](https://protect-eu.mimecast.com/s/x6asCzXkjiOvYXAsngj65?domain=journals.plos.org) for published examples.

Why was this study done?
Line 49 Please add the heading “Author Summary” before the sub-headings
Please ensure under each sub-heading, individual statements follow bullet-points.
Added

What did the researchers do and find?
This section is rather long. Please trim to no more than 4 individual bullet points

Now shortened shortened to 4 bullet points

Line 80: please revise the sub-heading to read “What do these findings mean?”

Changes made as requested

INTRODUCTION
Lines 101 and 102: please only include non-proprietary names for vaccines

Proprietary names deleted and in discussion

Line 119: Sentence beginning “Given…” suggest moving to an appropriate part of the methods section
Now moved

METHODS and RESULTS
Lines 192: see statistical reviewer comments also, which we agree with, please provide brief details of your statistical methods here. For all observational studies, we ask that in the manuscript text you indicate:
(1) the specific hypotheses you intended to test,
(2) the analytical methods by which you planned to test them,
(3) the analyses you actually performed, and
(4) when reported analyses differ from those that were planned, transparent explanations for differences that affect the reliability of the study's results. If a reported analysis was performed based on an interesting but unanticipated pattern in the data, please be clear that the analysis was data-driven.

We referred to the statistical plan in the protocol as we wanted to make it clear that this was a pre-planned analysis and provided it in Appendix 1, but the section underneath entitled “SCCS analysis” contained the key elements of the SCCS analysis extracted from the protocol that provided the information requested above. We have added a sentence to indicate that the additional age/gender breakdown for those under 40 years of age for myocarditis was carried out at the request of a reviewer as these were not mentioned in the protocol.

Lines 124: please include the number of residents (50 million as per the abstract)

Line 125: please define “outcomes of interest”
Line 159 onwards: please define PCR, LFT, UKHSA at first use
Line 166: please define NHS at first use
All done

Please remove governance, funding, data availability and conflicts of interest statements and include only in the relevant sections of the manuscript submission form. In the event of publication they will be complied as metadata
Done

As for the abstract, please quantify the main results with p values as well as 95% CIs. When reporting p values please report as p<0.001 (not .001) or where higher, the exact p value as p=0.002 (not .002), for example. Suggest modifying statistical reporting to read as follows: 3.23 (95% CI [1.85,5.64]; p= 0.0… or p<0.001…) and 6.06 (95% CI [2.95,12.47], p=0.00 or p<0.001). Please detail the statistical test (s) used to drive p values.

Reporting format with p values changed as requested. P values were derived with the Z-test which is now added to the methods section entitled “SCCS analysis”.

FIGURES
Please define all abbreviations including those used in the titles/captions in an appropriate footnote
Figure 1: in the caption please define “wk”
Figure 2: in an appropriate footnote, please indicate the meaning of the dots and lines
Figure 3: please include the year in the legend depicting the August date

Now added

TABLES
Please also see statistical reviewer comments which we agree with
As above, please define all abbreviations including those used in statistical reporting such as CI, for example
To help facilitate transparent data reporting where adjusted analyses are presented, please also include unadjusted analyses for comparison. In a caption/footnote please state which variables are adjusted for.

Unadjusted analyses are now included in the cohort analysis table in the supplementary appendix 3. The SCCS implicitly adjusts for individual level confounders and does not generate unadjusted estimates. Time period was included in the SCCS model as specified in the SCCS analysis section to allow for any temporal trends unrelated to the exposures of interest so we have now indicated this as a foot note in all SCCS tables.

Throughout where relevant please include a column for reporting p values instead of asterisks.
Please report p values as p <0.001 and where higher the exact p value. In an appropriate footnote, please detail the tests used to determine p values

P values now added to all tables. We have retained asterisks to highlight p values <0.01 and <0.001 to aid the reader identify outcomes that meet our pre-specified p values and which we draw attention to in the text

Table 1: please see statistical reviewer comments (to include 95% CIs) which we agree with

We have added CIs to all tables apart from table 1. Since the data in table 1 is from the whole population of England and are not estimates from a sample of the population it is not appropriate to show CIs. We have pointed this out to other journals in which data from the entire population in England has been shown and for which a request for RIs was made and it has been accepted (see for example table 1 *Characteristics of Persons Tested for SARS-CoV-2 in England, According to Test Positivity or Negativity and Variant*  in

[Covid-19 Vaccine Effectiveness against the Omicron (B.1.1.529) Variant | NEJM](https://protect-eu.mimecast.com/s/zqyzCx1g4cPpJrBc8WLp7?domain=eur01.safelinks.protection.outlook.com)

Table 3: please use commas to separate upper and lower confidence limits instead of hyphens as these can be confused with the presentation of negative values, and to ensure consistency in statistical reporting. Please check and amend where necessary including supplementary files

All now amended to comply with above request.

SUPPLEMENTARY FILES Throughout where relevant please include a column in the tables for reporting p values (instead of asterisks). Please indicate in a footnote the statistical test(s) used to determine them
Please ensure all abbreviations used for statistical reporting are appropriately defined including CI
Figure S3A – suggest full terms instead of myo- or peri-

We have now removed this shorthand notation from the tables, figs and text and - p values are shown for all tables. Fig S3A now removed as points covered in new para 2 in discussion
REFERENCES
For in-text reference callouts please ensure citations are separated by commas without spaces. For example, line 364, “…proposed [21, 22, 23].” Should read “…proposed [21,22,23].”

Done

Comments from the reviewers:

**Statistical reviewer:**

Major Comments

**Review of ‘Risk of myocarditis after a COVID-19 mRNA vaccine booster following homologous or heterologous priming in those with and without prior SARS-CoV-2 infection; an observational database study in England’**

The manuscript reports on the risk of hospital admission for myocarditis/pericarditis following the administration of booster doses with either of the two FDA-approved mRNA vaccines, after priming with adenovirus or mRA vaccines. This topic should be of broad appeal to the PLOS Medicine audience.

The manuscript is clearly written and the findings are important. The robustness of the study’s findings was demonstrated through consistency of the SCCS and retrospective cohort analysis results. The authors also assessed the SCCS model assumptions of no long-term event dependence. A sensitivity analysis was performed and limitations of the study were well described.

Some comments and suggestions follow.

**Major Comments**

The multiplicity adjustment is not well justified and appears rather arbitrary. How was the alpha-level of 0.001 selected? The multiple testing spanned time intervals, demographic subgroups, and endpoints (pericarditis, myocarditis, and myocarditis and/or pericarditis). It was not clear how many multiple tests were conducted and whether this 0.001 alpha level adequately controls the family-wise error rate (FWE) or the false-discovery rate (FDR) at a given level. I would recommend that the authors apply a method like Holm-Bonferroni to compute adjusted p-values that control the FWE at 0.05.

We acknowledge that many tests have been done and the number has increased following conduct of the additional ad hoc analyses suggested by the reviewers. The use of p<0.001 is in the original statistical analysis plan because we were aware there were a large number of groups and intervals were being assessed and wanted to reduce the false discovery rate. We prefer the approach of p-value cut-offs as giving indications of levels of evidence along with effect sizes. Adjusting p-values is not straight forward because many tests are correlated and there is a mix of primary analysis outcomes and additional analyses/stratifications, so not all tests are equal. We therefore prefer to keep the original planned approach and use the discussion to weigh up the level of evidence. We trust this approach is acceptable

A brief section on the statistical analysis methods used should be included in the manuscript rather than simply referring to the analysis plan in the protocol.

We referred to the statistical plan in the protocol as we wanted to make it clear that this was a pre-planned analysis and provided it in Appendix 1, but the section underneath entitled “SCCS analysis” contained the key elements of the SCCS analysis extracted from the protocol that provided the information requested above. We have added a sentence to indicate that the additional age/gender breakdown for those under 40 years of age for myocarditis was carried out at the request of a reviewer as these were not mentioned in the protocol or the SCCS analysis section of the paper.

Recommend providing 95%CIs on pg5 lines 74, 78

We have now removed the mention of attributable risk estimates from this section at the request of the editors to shorten it to just 4 bullet points.

Appendix S2: Please explain which analyses suggest that the 21-day pre-exposure window length is appropriate.

Relative incidences with varying pre-exposure windows are plotted in Appendix S2, with explanation and findings; RIs change very little with pre-exposure intervals longer than 21 days. This was also mentioned in the SCCS analysis section of the methods where we say as follows:

*The model accounted for short-term dependence, with the appropriate length of the pre-exposure period investigated; long-term event dependence was also investigated (S2 Appendix). These analyses indicated a pre-vaccination period of 21 days would be adequate to account for short-term event dependence and that there was no major concerns regarding longer-term dependence.*

We did not show the number of cases plotted by time period before and after vaccination in Appendix 2 as they can be misleading because this takes no account of person-time.

Pg 12, Line 261: Good to show no overlap in 95%CIs to back up the statement.

Since the data in table 1 is from the whole population of England not estimates from a sample of the population we do not show CIs. We are sorry if this wasn’t clear from the table heading which we have now amended.

Pg 15, Line 333: Add 95%CIs to AR estimates for admission.

Now added and to the table showing ARs

Table 1: Add 95%CIs to columns with SUS and ECDS risk estimates.

Since the data in table 1 is from the whole population of England not estimates from a sample of the population we do not show CIs. We are sorry if this wasn’t clear from the table heading which we have now amended.

Table 3: Add column of p-values.

Now added but table relegated to Appendix 3 (numbered Appendix S3G) at request of other reviewers

Minor Comments

Pg 9, line 173: It would be helpful to explain why those who received their 3rd dose before 1 Sept 2021 and those who received their 1st dose before 8 Dec 2020 were excluded. What is the significance of those dates?

Thank you for this comment. Routine vaccination in England only started on 8^th^ December 2020 and the booster programme started around mid September 2021 so primary doses given before 8th December or booster doses before September may not be part of the routine programme eg to individuals taking part in clinical trials. We have now clarified the rationale for these exclusions in the paper.

Abstract and Table 1: provide exact age range of study cohort: 12-x years. Could add a footnote to Table 1 noting the age of oldest person.

Now added- the oldest person in the study population was 101 years.

Appendix S2, paragraph 5, second sentence, add “generally”: mRNA dose 1 and2 effects were generally a little higher”

Now added

Figs S2A, S2B, Table S2A, S2B: Use same vaccine group labels as main manuscript. Define d1, d2 as dose 1, dose 2. Add p-values to tables.

Tables changed as requested. The figure in the main paper also uses the AZ, PF and MD notation due to space limitation on the axis labels but we provide the key to this notation in the figure caption. We have therefore added this as a footnote to the two figure captions in Appendix 2 and also indicated what d1 etc means which we trust suffices.

Pg 10, line 210: Would it be better to call it a secondary or exploratory analysis here rather than a sensitivity analysis? It seems to be addressing a separate question than checking on the robustness of the primary analysis. Lines 211-214 do seem to appropriately describe a sensitivity analysis to check for ascertainment bias.

Thank you for this suggestion. We do refer to both these analyses in the protocol and call then sensitivity analyses but agree that the first is better described as an exploratory analysis. We have therefore changed the main text as you suggest.

Figures 1-3 are very low resolution- quality should be improved. Please also use the same vaccine labels as main manuscript and use different colors or symbols instead of shades of grey to distinguish these.

Fig 3: Add 95%CIs to RI estimates.

Colours and 95% CIs now added– we removed fig 2 so Fig 3 is now Fig2 in the revised version

Reviewer #2: Thank you for the opportunity to review this manuscript. It was easy to read and the analysis appears to be appropriate. The question of myocarditis risk after booster doses is important. Reassuring to see that while there still is an effect, it appears to be attenuated. The addition of infection including breakthrough infections and re-infections adds valuable perspective to our current situation.

1) The main issue is the overlap with the Patone paper1, which is also an SCCS analysis of booster vaccination/infection in the same data. The Patone paper has follow-up among 13+ years of age from December 1, 2020, to December 15, 2021 compared to the this study which has follow-up among 12+ years of age from February 22, 2021 to February 6, 2022. But otherwise, it appears to be very similar with respect to data and analysis. It needs to be clarified in the manuscript what the key differences are and how this adds significantly to the Patone analysis.

Thank you for this comment. The Patone et al paper was published shortly before we submitted our paper to PLoS Med in September 2022 and after we had conducted the literature review for our paper. We therefore only became aware of it shortly after submission hence our omission of any mention of the Patone et al analysis. We have now ensured that differences between the Patone et al analysis and ours are highlighted in the introduction.

2) Another main issue is the combination of myocarditis and pericarditis into one outcome. Myocarditis and pericarditis background rates differ by age and sex, which means that it is difficult to advice public health policy from numbers based on a combined outcome. I suggest that they are analysed separately throughout - myocarditis results could be presented in text and pericarditis in supplementary.

Thank you for this suggestion which we have now implemented for the post-vaccine analyses and show myocarditis in the table in the main paper and just show the post-vaccination pericarditis outcomes in the appendix. For the infection analyses however the RIs are similar for both the myocarditis and pericarditis outcomes for the 1-27 day period so we have retained the combined outcome for the post infection table 3 (previously table 4) and for the attributable risk estimates in table 4 (previously table 5).

3) Abstract could be shortened (and made more readable) by reducing the number of results presented here - identify and focus on the key findings.
4) The introduction/discussion is outdated. Should be updated to include the newest studies on booster vaccination and myocarditis risk. (1-3)

Thank you for drawing our attention to the additional papers published recently which we have referenced in our revised discussion.

5) I would not include emergency care outcomes at all. I expect them to have poor validity. I would restrict the main analysis to hospital in-patients. Maybe place the emergency care outcomes in supplementary only?

We have now relegated all emergency care outcomes to the Supplementary appendix as suggested. However we do feel that this is a useful additional data source especially for pericarditis for which in England hospital admission, particularly in healthy young adults without co-morbidities, is rare. This is reflected in Table 1 which shows 3401 emergency care consultations in <40 year olds for pericarditis but only 561 admissions. As a consequence many of the post-vaccination risk intervals in the hospital admissions analysis for pericarditis have n<2 for which RIs were not estimated. Emergency care consultations for pericarditis and myocarditis showed a similar pattern of elevated risks post-vaccine as admissions for myocarditis in adults aged 16-39 years which is supportive of the specificity of the diagnoses in the ECDS data set. We therefore feel that this data sources is useful and have now included a new para (para 2) in the discussion to justify its inclusion.

6) Lines 189-190: Please elaborate on why follow-up was not censored at event.

The cohort was extremely large as it comprised over 50 million individuals and since the outcome even was very rare it was computationally simpler to not censor the small number of individuals with the outcome of interest at their event date but to use a common end date for the cohort. This enabled the analysis to be completed more rapidly as the results were urgently required by our vaccine advisory committee. We have expanded the existing sentence as follows:

*As outcome events were rare, for computational simplicity and to allow more rapid delivery of results for policy makers person time was censored at 6th February 2022 not at event date.*

attributable risk results should ideally also be presented with 95% CIs to reflect the statistical precision of the estimates.

Now added

9) Can you comment on the impact of the testing policy / capture of infections in England? E.g. attributable risks will be dependent on background rates of infections. How many infections do you estimate that you have captured by tests in England?

Thank you for this suggestion. As the AR estimates post-infection are just for those with a laboratory-confirmed infection we now make this clear in table 4 (previously table 5) and also include a brief comment in the limitations para in the discussion that lab confirmed infections will underestimate the true number in the population so precluding a direct comparison with the post-vaccination risk. Prompted by your question we did think a bit more about the extent to which the laboratory confirmed cases underestimated the actual number of infections in the population. Based on testing English blood donors for N antibodies using samples taken up to the end of January 2022, it was estimated that the overall population weighted (by age group, sex and NHS region) antibody prevalence among blood donors aged 17 years and older in England was 28.6% (95% CI 27.8% - 29.4%) using the Roche N assay (see page 47 in the UKHSA report available at <https://protect-eu.mimecast.com/s/pcAtCzXkjiOzNpQc43ux0?domain=eur01.safelinks.protection.outlook.com>.

The comparable prevalence at the end of January 2021 was 10% so a difference of 18.6% which in a population of 50 million would amount to some 10 million infections whereas the total confirmed in the study period was around 14 million. The sero-epidemiological data will underestimate infections however as N antibodies wane fairly rapidly, reinfections will not add to the existing N antibody prevalence and samples in January will reflect infections up to the end of December due to the lag in seroconversions to the N antigen so will miss much of the Omicron wave with its high infection and reinfection rate. In England as elsewhere there was (until end March 2022) widespread community testing which was free to all including contacts of known cases plus mandatory regular testing of health and social care workers so perhaps this high capture rate of all infections isn’t too surprising.

1. Patone M, Mei XW, Handunnetthi L, et al. Risk of Myocarditis After Sequential Doses of COVID-19 Vaccine and SARS-CoV-2 Infection by Age and Sex. Circulation. 2022;146:743-754. doi:10.1161/circulationaha.122.059970
2. Naveed Z, Li J, Spencer M, et al. Observed versus expected rates of myocarditis after SARS-CoV-2 vaccination: a population-based cohort study. CMAJ. 2022;194(45):E1529-E1536. doi:10.1503/CMAJ.220676
3. Stéphane Le Vu A, Bertrand M, Jabagi MJ, et al. Risk of Myocarditis after Covid-19 mRNA Vaccination: Impact of Booster Dose and Dosing Interval. medRxiv. Published online August 1, 2022:2022.07.31.22278064. doi:10.1101/2022.07.31.22278064


Reviewer #3:

This is a very well written paper on an important topic. Also, the statistical analyses used are adequate. However, there are some concerns. My main concern is the combination of myocarditis and pericarditis combined. Apart from, maybe, diluting, the effect of vaccination on risk of vaccination in young males it also raised the question whether the data sources actually capture all cases?

The Title is misleading as the presented comparison is actually on myocarditis and pericarditis combined.

Title now revised and also to reflect other reviewers’ comments.

Page 9, 197 exposure and short-term event independency. You did assess this by inclusion of a pre-exposure period. Would be nice to just see a plot of number of days since myocarditis patients were vaccinated? I would guess very few cases would actually be vaccinated within 1-3 weeks after an event?

Thank you for this comment – the plot is shown below. However plots of the interval between vaccination and events can be misleading because they take no account of person-time, hence we will not include this in the paper or supplementary material. Relative incidences with varying pre-exposure windows are plotted in Appendix S2, with explanation and findings; RIs change very little with pre-exposure intervals longer than 21 days which was the justification we cited for using the 21 day pre-exposure window.

Page 10, 209. A bit strange and uncommon in epidemiology to define "strong" evidence based on the p-value. :-)

Yes this was badly worded. We have now changed this to “strong statistical evidence of an association” which was the intention behind the wording as indicated in section 4f of the protocol.

Results
Page 12, 253/ Table 1. Myocarditis and pericarditis are different diseases and their incidence vary by age. Myocarditis is more common in younger ages and pericarditis in older. Most studies on myo/peri risk after vaccination have done separate analyses on these two conditions. Why was that not done here? Presentation of incidence rates by age as in table1 for these conditions combined is not informative. Suggest to present incidence by age and sex for myo and peri separately (and peri can be in supplement). And subsequently all analyses separately for myo/peri ( I.e., suggest S3C and D as main tables).

Thank you for this suggestion. We now show post-vaccination outcomes for myocarditis separately from pericarditis though have retained the joint outcome for post-infection analyses as the risk of myocarditis and pericarditis after SARS-CoV-2 infection is similar.

Also, is there any information on the validity of these diagnoses in the registers used? This in regards to the (much) lower incidence found in your data compared to others.

The diagnoses coded as myocarditis in the SUS data set that we used were not validated against case note review (which we acknowledge in the limitations paragraph) nor were they in the Nordic study by Karlstad et al. However despite the differences in background rates in admissions for myocarditis and pericarditis between the Nordic study and our own, the findings in terms of elevated risk by vaccine product and dose in the primary series between our studies are similar. In relation to differences in background rates between the English and Nordic studies the latter used outpatient contacts for myocarditis as well as inpatient admissions. We were unable to find a breakdown in the Nordic study of the incidence rates by outpatient consultation and hospital admission so could not compare directly with the inpatient rates in our study. In Israel the Mevorach et al study only used hospital admitted cases but stated that any patient with a diagnosis of myocarditis would be admitted. Also the Israeli study used active surveillance methods in which clinicians were alerted to the potential association with vaccination and were requested to report such cases. The paragraph dealing with differences in incidence rates between studies is now expanded to provide this additional information. We do not consider that differences in background rates in different settings raises questions about the validity of the diagnoses in our study.

Also, the risk has been shown to be highest in 16-24 (rather than in 25-39). Would suggest to also present this age group, (16-24, 25-39).

Thank you for this suggestion. We have now carried out this sub-analysis which does show higher RIs for myocarditis in 16-24 year olds than 25-39 year olds, particularly for the Moderna vaccine. We have included this additional analysis in the supplementary appendix 3 and noted it in the discussion.

Death. Is it only possible to assess death within the first week (0-6 days) and not for eg after 28 days? Also, presentation of deaths (as all results!) should be separately for myo and peri. Do you not find any death in young (below 40) after "vaccine-induced" myocarditis in your data?

We reported deaths during admission or within a week of discharge as being most likely to be attributable to the cardiac condition. We then used this measure to compare the fatality rate for unvaccinated cases (the background) with those admitted within 0-6 days of vaccination as it is this interval in which there is evidence of an excess compared with the background rate. We have reworded this to hopefully make it clearer that we weren’t just counting deaths within the first 0-6 days. Of the three deaths within 0-6 days of a vaccine that we report all had a diagnosis of myocarditis which we have added to the discussion and only one was a young adult but with other co-morbidities – hence our statement that we found no deaths in healthy young adults during admission or within a week of discharge among those with onset of myocarditis or pericarditis within 6 days of vaccination.

Page 17, 387 paragraph comparing with other studies. Would suggest to compare effect estimates for the most important groups ( i.e., myocarditis in young men). As stated now you appear to find one fourth of the overall incidence of myo and peri as found in the Nordic study. To better understand the validity of your data I would suggest presenting numbers for young men and myocarditis only (not combined with pericarditis).

See response above in relation to comparison of background rates. We do not believe that such differences call into question the validity of our data. We have conducted a further sub analysis as requested splitting by gender those with myocarditis aged 16-39 years and see as expected from the gender and age split in table 2 that most of the excess cases are in males.

Conclusion.
Page19, 449. Would avoid mentioning the comparison of outcome after vaccination with outcome after a positive covid test as the latter, as you also mention, is (highly) dependent on testing strategy (eg. When admitted to hospital). The fewer less severe tested the "higher" the risk of outcome after covid. The benefit-risk analyses is not based on comparing risk of myo after vaccination with that after covid. Hence, would avoid supporting such comparisons.

Thank you for this comment – we have amended the conclusions accordingly.

Tables. Would suggest removing the "two-star" "three-star" significance throughout :-)
Table 5. I guess, column heading should be " attributable… per million vaccinations" for the data regarding the vaccinees.

We now show actual p values for all outcomes but have retained the **and *** notation to allow readers to easily identify those outcomes that meet our pre-specified P values. Thank you for pointing out or mistake in the column heading for table 4 (now 5).

Another recent reference that might be of interest:
Booster Vaccination with SARS-CoV-2 mRNA Vaccines and Myocarditis Risk in Adolescents in and Young Adults: A Nordic Cohort Study of 8.9 Million Residents | medRxiv

Thank you – this was posted after we submitted our paper but have now included mention of it in our paper.
