# Supplementary material for: Risk of myocarditis and pericarditis after a COVID-19 mRNA vaccine booster and after COVID-19 in those with and without prior SARS-CoV-2 infection: A self-controlled case series analysis in England
Source: PLoS Med. 2023 Jun 7;20(6):e1004245. doi: 10.1371/journal.pmed.1004245 (PMC10286992; doi:10.1371/journal.pmed.1004245)
Supplement: Attachment — Submitted filename: Re-review_22-03092R1.docx [file pmed.1004245.s007.docx]

**Re-review of ‘Risk of myocarditis after a COVID-19 mRNA vaccine booster following homologous or heterologous priming in those with and without prior SARS-CoV-2 infection; an observational database study in England’**

The authors have responded to the majority of my comments. There remains a lack of clarity on the primary objectives/analyses and formal multiplicity adjustment for these. It is critical that the authors clearly lay out in the manuscript itself the primary and secondary objectives and the associated hypothesis tests considered. The mix of primary analysis outcomes and additional analyses and stratifications could be better structured to first highlight the primary analyses and then describe the secondary, exploratory, and ad-hoc analyses. Multiplicity adjustments can be done within each tier of analyses rather than using a single threshold of p<0.001 which may be too conservative or too liberal depending how many tests were performed. At a minimum, it would be helpful for the authors to note how many hypothesis tests were performed.
