# Supplementary material for: Risk of myocarditis and pericarditis after a COVID-19 mRNA vaccine booster and after COVID-19 in those with and without prior SARS-CoV-2 infection: A self-controlled case series analysis in England
Source: PLoS Med. 2023 Jun 7;20(6):e1004245. doi: 10.1371/journal.pmed.1004245 (PMC10286992; doi:10.1371/journal.pmed.1004245)
Supplement: Attachment — Submitted filename: rev plos med.docx [file pmed.1004245.s008.docx]

Table 1: please see statistical reviewer comments (to include 95% CIs) which we agree with Author response: *We have added CIs to all tables apart from table 1. Since the data in table 1 is from the whole population of England and are not estimates from a sample of the population it is not appropriate to show CIs. We have pointed this out to other journals in which data from the entire population in England has been shown and for which a request for RIs was made and it has been accepted (see for example table 1 Characteristics of Persons Tested for SARS-CoV-2 in England, According to Test Positivity or Negativity and Variant in Covid-19 Vaccine Effectiveness against the Omicron (B.1.1.529) Variant | NEJM*

[Minor comment: I agree with the authors. But, sometimes CI can be motivated by seeing national data as a sample in time ( i.e. it is not a sample of the population, but the population differs by years, hence it could sometimes be useful with CI)]

Page 9, 197 exposure and short-term event independency. You did assess this by inclusion of a pre-exposure period. Would be nice to just see a plot of number of days since myocarditis patients were vaccinated? I would guess very few cases would actually be vaccinated within 1-3 weeks after an event?

*Thank you for this comment – the plot is shown below. However plots of the interval between vaccination and events can be misleading because they take no account of person-time, hence we will not include this in the paper or supplementary material. Relative incidences with varying preexposure windows are plotted in Appendix S2, with explanation and findings; RIs change very little with pre-exposure intervals longer than 21 days which was the justification we cited for using the 21 day pre-exposure window.*

Comment: It is clear from the plot that individuals are not at all that likely to be vaccinated within 14 days of a myocarditis event. (Do not understand the comment on person-time. The study, as I understand, does not really take individual person-time into account e.g. persons are not censured after an event of at time of a vaccine-schedule that is not studied?)

Page 10, 209. A bit strange and uncommon in epidemiology to define "strong" evidence based on the p-value. :-)

*Yes this was badly worded. We have now changed this to “strong statistical evidence of an association” which was the intention behind the wording as indicated in section 4f of the protocol.*

Minor Comment: my comment was more on that “strong evidence” should more be based on the magnitude of the association, dose-response etc than on a p-value. But, it is apparent the Journal wants p-values.

Also, is there any information on the validity of these diagnoses in the registers used? This in regards to the (much) lower incidence found in your data compared to others.

*Author response: The diagnoses coded as myocarditis in the SUS data set that we used were not validated against case note review (which we acknowledge in the limitations paragraph) nor were they in the Nordic study by Karlstad et al. However despite the differences in background rates in admissions for myocarditis and pericarditis between the Nordic study and our own, the findings in terms of elevated risk by vaccine product and dose in the primary series between our studies are similar. In relation to differences in background rates between the English and Nordic studies the latter used outpatient contacts for myocarditis as well as inpatient admissions. We were unable to find a breakdown in the Nordic study of the incidence rates by outpatient consultation and hospital admission so could not compare directly with the inpatient rates in our study. In Israel the Mevorach et al study only used hospital admitted cases but stated that any patient with a diagnosis of myocarditis would be admitted. Also the Israeli study used active surveillance methods in which clinicians were alerted to the potential association with vaccination and were requested to report such cases. The paragraph dealing with differences in incidence rates between studies is now expanded to provide this additional information. We do not consider that differences in background rates in different settings raises questions about the validity of the diagnoses in our study.*

Comment:

Danish ICD codes of myocarditis, as part of the Nordic study, have been validated (Ref Sundbøll  J, Adelborg  K, Munch  T,  et al.  Positive predictive value of cardiovascular diagnoses in the Danish National Patient Registry: a validation study.   *BMJ Open*. 2016;6(11):e012832. doi:[10.1136/bmjopen-2016-012832](http://dx.doi.org/10.1136/bmjopen-2016-012832)). Also, the Nordic study did not include outpatient cases: “*We defined incident outcome events as the date of first hospital admission for myocarditis or pericarditis from December 27, 2020, onward. The primary outcome was a main or secondary diagnosis of myocarditis at discharge from inpatient hospital care.* [SARS-CoV-2 Vaccination and Myocarditis in a Nordic Cohort Study of 23 Million Residents | Cardiology | JAMA Cardiology | JAMA Network](https://jamanetwork.com/journals/jamacardiology/fullarticle/2791253) “

If the authors see no problem in large differences in background rates do they have an explanation why rates of myocarditis are much lower compared to e.g., the Nordic countries?

I agree that it is of less importance when discussion relative measures. But, measures of absolute risk, incidence rate, excess risk are dependent on background risks. The authors do acknowledge this is the discussion. But, their explanation why they find lower rates in not really reassuring.

Methods page 7 line 141:

“*The study population comprised the resident population of 50 million individuals in England aged 12 years and older on the 31st August 2021. Dates of admission for the myocarditis or*

*pericarditis were from 22nd February 2021 to 6th February 2022.*” Is 31 Aug 2021 the correct date? Cannot see how you can have the study pop on a later date than first outcome (22 Feb 2021)?

Page 7, line 146:

*“Given the generally mild nature of vaccine-associated myocarditis [14] we analysed cases presenting in emergency care settings as well as those admitted to hospital.*”

Well, if the authors want to state anything on the severity of vaccine-associated myo compared to myo of other causes I would suggest reading of some more detailed references:

Witberg G, Barda N, Hoss S, Richter I, Wiessman M, Aviv Y, et al. Myocarditis after Covid-19 vaccination in a large health care organization. N Engl J Med 2021;385: 2132–2139. 2.

Mevorach D, Anis E, Cedar N, Bromberg M, Haas EJ, Nadir E, et al. Myocarditis after BNT162b2 mRNA vaccine against Covid-19 in Israel. N Engl J Med 2021;385: 2140–2149

[Clinical outcomes of myocarditis after SARS-CoV-2 mRNA vaccination in four Nordic countries: population based cohort study | BMJ Medicine](https://bmjmedicine.bmj.com/content/2/1/e000373) <https://bmjmedicine.bmj.com/content/2/1/e000373>

Page 19, line 444

“This may be attributable in part to study design as outpatient and inpatient myocarditis cases were included in the Nordic study”

This is not true. The Nordic study states: “*We defined incident outcome events as the date of* ***first hospital admission*** *for myocarditis or pericarditis from December 27, 2020, onward. The primary outcome was a main or secondary diagnosis of myocarditis at* ***discharge from inpatient hospital care****.* [SARS-CoV-2 Vaccination and Myocarditis in a Nordic Cohort Study of 23 Million Residents | Cardiology | JAMA Cardiology | JAMA Network](https://jamanetwork.com/journals/jamacardiology/fullarticle/2791253) “

Page 23, line 518. Last sentence in conclusion. “*The rarity and benign outcome of vaccine attributable cases is reassuring*.”

Well, here the authors discuss their detected incidence rates, which are (much) lower than other studies, and also discuss the severity of the outcome of which they have no supporting data except case fatality within one week. Is this sentence really needed?

Happy to see the stratification by age and sex (e.g., males 16-24) now being presented. In general, all studies on myo/peri should be stratified by sex and age as the incidence varies substantially.
